# Chemical Composition and Bioactive Properties of *Camellia oleifera* C. Abel Leaves

**DOI:** 10.3390/molecules30183661

**Published:** 2025-09-09

**Authors:** Jun Chen, Lilin Xiang, Deliang Qiao, Changli Min, Li Zhang, Xuejun Wang

**Affiliations:** 1 College of Biotechnology and Pharmaceutical Engineering, West Anhui University, Lu’an 237012, China; 02000179@wxc.edu.cn (J.C.); lilin.xiang@rainbowfish11000.com (L.X.); qiaodl@wxc.edu.cn (D.Q.); mcl0917@163.com (C.M.); 2 Analytical and Testing Center, West Anhui University, Lu’an 237012, China; 02000081@wxc.edu.cn

**Keywords:** *Camellia oleifera* C. Abel leaves extracts, flavonoids, polyphenols, antioxidant, tyrosinase, cell proliferation

## Abstract

*Camellia oleifera* C. Abel is an economically important oilseed crop. This study aimed to investigate the chemical composition and bioactive potential of its leaf extracts, an underutilized by-product, for cosmetic and pharmaceutical applications. Extracts of *C. oleifera* leaves were prepared using three solvents (water, 50% ethanol, 95% ethanol) via ultrasonication. The total polyphenol and flavonoid contents were quantified, and key bioactivities, including antioxidant capacity, tyrosinase inhibition, and effects on cell proliferation, were evaluated. The 50% ethanolic extract exhibited the highest total polyphenol (337.24 ± 1.94 GAE/g extract) and total flavonoid (189.23 ± 1.12 mg RE/g extract) contents. This extract also demonstrated superior antioxidant activity, with an IC_50_ of 28.10 ± 0.46 μg/mL for DPPH scavenging and an ORAC value of 2651.54 ± 112.41 μmol/g. Nine compounds were isolated and identified, comprising flavonoids (**1**–**3**) and polyphenols (**4**–**9**). Compound **1** showed the strongest DPPH scavenging activity with IC_50_ of 24.19 ± 0.07 μM. Compound **9** significantly stimulated HaCaT cell proliferation (169.30 ± 2.17%), while compound **2** promoted the growth of HFF-1 cells (129.36 ± 2.81%). These results highlight the potential of *C. oleifera* leaves as a valuable source of bioactive compounds for cosmetic and pharmaceutical applications.

## 1. Introduction

**Figure 1:**
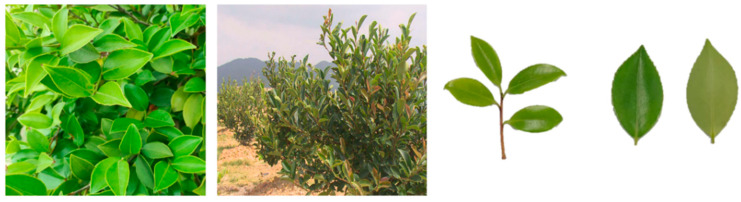
Images from the studied *Camellia oleifera* C. Abel leaves extracts.

*Camellia oleifera* C. Abel, a species within the Theaceae family, is an economically important tree native to China, primarily cultivated for its seeds to produce tea seed oil. However, the leaves of *C. oleifera* (Figure 1) represent an underutilized by-product with significant potential. In traditional medicine, these leaves have been used for their astringent and hemostatic properties, applied in the treatment of ailments such as nosebleeds, skin ulcers, pruritus, and abscesses [1]. Contemporary pharmacological studies have revealed that *C. oleifera* leaves possess anticancer and antibacterial properties [2,3]. Furthermore, research indicates that extracts from these leaves can inhibit cysteine proteases, thereby potentially extending cellular lifespan. Coupled with their antioxidant and free radical-scavenging capabilities [4,5], *C. oleifera* leaf extracts show promise in combating wrinkles and other signs of skin aging, in addition to exhibiting anti-inflammatory and potential weight management effects.

The growing consumer preference for natural ingredients in cosmetics has driven research into plant extracts. Common botanical sources include rose, ginseng, grape seed, aloe vera, and rosemary, which are rich in bioactive compounds with established anti-aging, moisturizing, and whitening effects [6,7]. Notably, *C. oleifera* leaf extracts are listed in the 2021 Directory of Cosmetic Ingredients in China, providing a firm regulatory foundation for their use in cosmetic formulations [8]. Modern pharmacological studies have further confirmed that *C. oleifera* leaves possess anticancer and antibacterial activities [9].

*C. oleifera* leaves contain numerous bioactive compounds, including polyphenols, flavonoids, terpenoids, saponins, alkaloids, and other components [10,11]. Despite this, the comprehensive phytochemical profile of the leaves remains relatively unexplored. Early investigations led to the isolation of flavonoids such as quercetin, quercetin-3-O-rhamnoside, and quercetin-3-O-α-glucosyl-(6→1)-α-rhamnoside. Subsequent studies employing thin-layer chromatography, mass spectrometry, and NMR techniques identified additional compounds, including 1-(3′,5′-dimethoxy) phenyl 2-[4′-O-β-D-glucosyl-(6→1)-O-α-L-rhamnosyl]-phenylethane, quercetin-3-O-α-L-rhamnoside, and dihydrokaempferol were identified in the *C. oleifera* leaves extracts [12,13]. Other isolated and identified constituents comprise quinic acid, carotene, kaempferol, quercetin-3-O-β-D-glucoside, and quercetin-3-O-β-D-galactoside [14]. Methanolic extracts of the leaves have demonstrated potent inhibitory effects on 5α-reductase (5α-R), suggesting potential applications in treating conditions like androgenic alopecia and acne [15]. Additionally, polyphenols from *C. oleifera* have been shown to significantly alter the metabolic processes of soluble proteins in plant pathogenic fungi, offering a theoretical basis for eco-friendly plant disease control.

During annual pruning and early growth stages, significant quantities of *C. oleifera* leaves are typically discarded or burned, leading to resource waste and environmental concerns. The limited research on the chemical constituents and pharmacological activities of these leaves highlights a critical knowledge gap.

This study aims to bridge this gap by systematically isolating and identifying the primary bioactive chemical constituents from *C. oleifera* leaves and evaluating their relevant bioactivities. The overarching goal is to lay a foundation for the potential pharmacological application and industrial valorization of this abundant agricultural by-product.

## 2. Results and Discussion

### 2.1. Total Flavonoid and Phenolic Content of Extracts

The total flavonoid (TF) and phenolic (TP) contents of the three extracts varied significantly (Table 1). The 50% (*v*/*v*) ethanolic extract (HEE) yielded the highest contents of both TF (189.23 ± 1.12 mg RE/g extract) and TP (337.24 ± 1.94 mg GAE/g extract). This can be attributed to the optimal polarity of 50% ethanol for efficiently extracting a broad range of polar phenolic compounds and flavonoid glycosides. In contrast, the aqueous extract (WE) exhibited lower TF and TP contents (120.52 ± 0.62 mg RE/g extract and 146.16 ± 1.89 mg GAE/g extract, respectively), while the 95% (*v*/*v*) ethanolic extract (EE) showed intermediate levels (160.60 ± 1.08 mg RE/g extract and 160.58 ± 0.78 mg GAE/g extract, respectively). Consequently, the rank order for both TF and TP content was: 50% ethanolic extract > 95% ethanolic extract > aqueous extract.

The selection of extraction parameters and solvents was guided by the dual objectives of achieving high efficiency and ensuring suitability for potential industrial applications, particularly in the cosmetic and pharmaceutical sectors. With a focus on developing a green and sustainable extraction process, ethanol and water were prioritized over conventional organic solvents like methanol. This decision was based on several key considerations: (1) Safety and Regulatory Compliance: Ethanol is classified as a Class 3 low-toxicity solvent by ICH Q3C guidelines and is compliant with EU Cosmetics Regulation (EC 123/2009) [16], avoiding the neurotoxic risks associated with methanol and simplifying the safety profile of the final extract for topical applications [17]. (2) Industrial Scalability and Environmental Impact: Ethanol is generally recognized as safe (GRAS), easier to recycle in large-scale processes, and presents a lower environmental burden compared to methanol, aligning with the principles of green chemistry [18]. (3) Consumer Acceptance: Solvent residues are a key concern for natural products; using ethanol and water ensures the final product is more readily acceptable to consumers.

The extraction conditions (40 °C, 1:10 solid-to-solvent ratio, and 30 min ultrasonication for 3 cycles) were selected based on a standardized conventional protocol widely used for the extraction of polyphenols and flavonoids. This approach prioritized reproducibility and the establishment of a robust baseline for comparing solvent efficacy over complex optimization at this initial stage. Consequently, this study systematically evaluated the impact of three solvents with varying polarities—water (WE), 50% ethanol (HEE), and 95% ethanol (EE)—on the yield, chemical composition, and bioactivity of *C. oleifera* leaf extracts.”

### 2.2. Chemical Composition of C. oleifera Leaves Extracts

**Figure 2:**
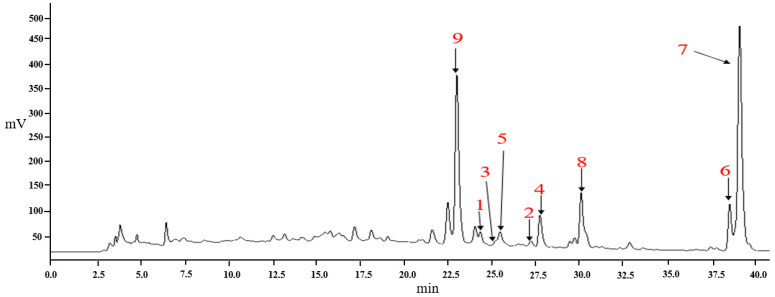
HPLC chromatograms of *Camellia oleifera* C. Abel leaves extracts. *X*-axis: Retention time (min); *Y*-axis: Absorbance (mV).

**Figure 3:**
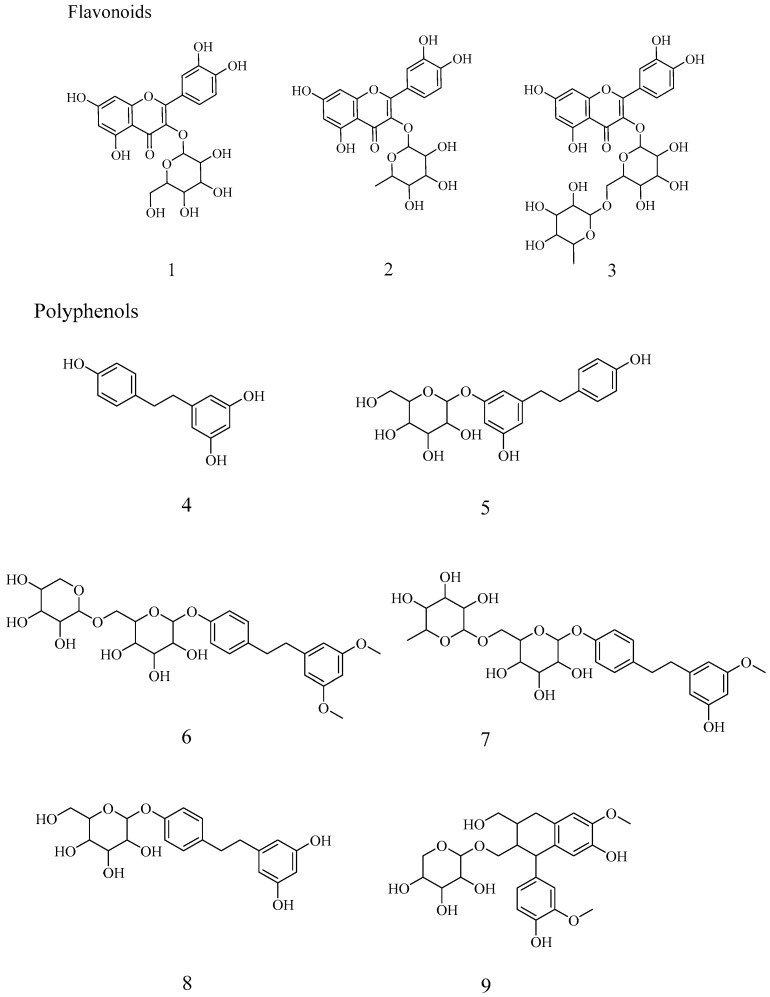
The structure of compounds.

The HPLC fingerprints of *C. oleifera* leaf extracts are shown in Figure 2. Peaks were identified by comparing their retention times and UV spectra with those of authentic standards, where available. Structural elucidation was further confirmed by NMR and MS analyses. A total of nine compounds were identified (Table 2, Figure 3), including flavonoids (**1**–**3**) [19,20,21] and polyphenols (**4**–**9**) [12,22,23,24].

These results demonstrate that *C. oleifera* leaves are a rich source of diverse secondary metabolites, with flavonoids and polyphenols constituting major classes. The established HPLC fingerprint, complemented by spectral identification, provides a robust method for characterizing the chemical composition of these extracts, facilitating future bioactivity studies and application development.

### 2.3. Antioxidant Activity

**Figure 4:**
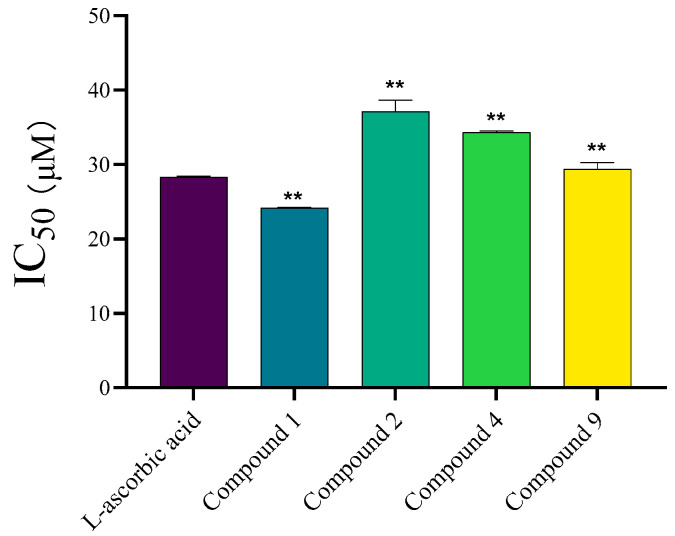
IC_50_ values for DPPH radical scavenging activity of isolated compounds from *Camellia oleifera* C. Abel leaves and L-ascorbic acid (positive control). Data are presented as mean ± SD (*n* = 3); ** *p* < 0.01 vs. control.

The antioxidant capacity of plant extracts is crucial for their potential in combating oxidative stress-related skin aging and enhancing product stability in cosmetic formulations. The DPPH assay measures the antioxidant capacity of antioxidants in scavenging the stable free radical 2,2-diphenyl-1-picrylhydrazyl. The radical DPPH is reduced to its corresponding hydrazine upon reaction with a hydrogen donor. The DPPH assay is considered an effective and convenient method for evaluating the antioxidant activity of natural products. The IC_50_ values for DPPH radical scavenging by isolated compounds are visualized in Figure 4.

Most compounds isolated from *C. oleifera* leaves exhibited potent DPPH scavenging activity, albeit with significant variation. Compound **1** demonstrated the highest activity, with IC_50_ values of 24.19 ± 0.07 μM, respectively, which were lower than that of the positive control, L-ascorbic acid. This indicates superior radical scavenging potency for these specific flavonoids. In contrast, Compounds **3**,**5**,**6**,**7** and **8** exhibited weak activity, with less than 50% inhibition even at the highest tested concentration (500 μM), precluding accurate IC_50_ determination.

The greater antioxidant potency of compound **1** is consistent with the well-established structure–activity relationship of flavonoids. The flavonoid compound **1** demonstrated the highest activity, with IC_50_ values of 24.19 ± 0.07 μM, respectively, which were lower than that of the positive control, L-ascorbic acid. This indicates exceptional radical scavenging potency for these specific flavonoids. This potent activity is a canonical example of flavonoid antioxidant structure–activity relationship. It can be attributed to two key structural features: The ortho-dihydroxy (catechol) moiety in the B-ring, which confers high stability to the resulting radical intermediate through hydrogen bonding and enhances hydrogen-donating capacity. The 2,3-double bond in conjugation with a 4-oxo function in the C-ring, which allows for electron delocalization across the entire molecule, stabilizing the radical form and increasing reactivity. This well-established mechanistic rationale robustly explains why these structural motifs, both present in compound 1, are consistent with the observed high antioxidant activity [25].

Our findings align with previous studies on other plant extracts where these two compounds were identified as major contributors to overall antioxidant power [26]. The weak activity of the other compounds, particularly the non-flavonoid polyphenols, suggests that their radical scavenging mechanism may be less efficient or that their specific chemical structures are not optimized for the DPPH assay. The practical value of this finding is significant: compound **1** could serve as a marker compound for standardizing *C. oleifera* leaf extracts intended for use as natural antioxidants in cosmetics or functional foods to combat oxidative stress.

### 2.4. Tyrosinase Inhibition

Tyrosinase is a key enzyme in melanin synthesis, and its inhibition is a primary target for skin-lightening and whitening cosmetic products. Using L-tyrosine as a substrate and tyrosinase as a catalyst, the inhibitory effect of monomeric compounds in *C. oleifera* leaves extracts on the tyrosinase activity was determined [27]. The activity inhibition rates of each compound on tyrosinase are summarized in Table 3.

At an initial concentration of 100 μM, none of the compounds achieved a tyrosinase inhibition rate exceeding 50%. Compound **3** showed the highest inhibition (40.75 ± 2.73%), followed by compound **4** (37.99 ± 2.83%), while the remaining compounds exhibited inhibition rates below 32%. The compounds **4** and **5** were approximately half that of the potent positive control kojic acid (77.76%) at the same concentration (100 μM); these results indicate that the isolated monomeric compounds display only moderate tyrosinase inhibitory activity, with none demonstrating significant efficacy under the tested conditions.

In conclusion, *C. oleifera* leaf-derived monomeric compounds show limited potential as tyrosinase inhibitors, suggesting that their bioactivity may be more pronounced in other pathways (e.g., antioxidant or proliferative effects) rather than melanin biosynthesis regulation.

### 2.5. Molecular Docking Analysis

To further elucidate the potential binding modes and inhibitory mechanisms of the isolated compounds against tyrosinase, molecular docking studies were performed using the crystal structure of *Agaricus bisporus* tyrosinase (PDB ID: 2Y9X) [20]. Among the tested compounds, compound **3** and compound **4** were selected for detailed analysis based on their relatively higher tyrosinase inhibition rates (40.75 ± 2.73% and 37.99 ± 2.83% at 100 μM, respectively; Table 3).

**Figure 5:**
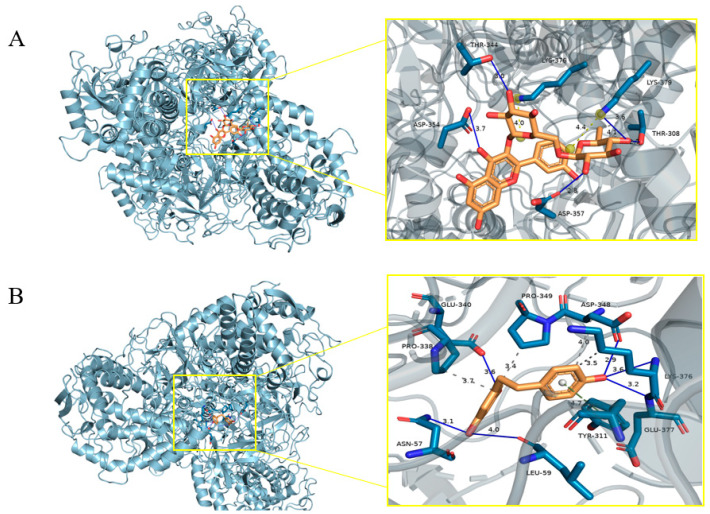
Molecular docking poses of (**A**) compound **3** and (**B**) compound **4** in the active site of Agaricus bisporus tyrosinase (PDB ID: 2Y9X).

The docking results revealed a significant disparity in binding affinity, with compound **3** exhibiting a markedly lower binding energy (−9.9 kcal/mol) compared to compound **4** (−7.7 kcal/mol). This computational prediction strongly correlates with their experimental inhibitory activities. As visualized in Figure 5A, compound **3** binds deeply within the active site pocket. Its stability is primarily conferred by the formation of three strong hydrogen bonds with the key residues THR-344, ASP-354, and ASP-357, which are located in close proximity to the catalytic copper core. This direct and strategic engagement with the enzyme’s central machinery effectively obstructs the substrate channel, providing a clear structural rationale for its potent inhibitory effect. In contrast, compound **4** (Figure 5B) adopts a binding pose that is more peripheral. Although it appears to engage a broader set of residues (ASN-57, LEU-59, GLU-340, ASP-348, LYS-376, GLU-377), the interactions are primarily clustered around the entrance of the active site.

Critically, despite compound **4** higher number of apparent interactions, its lower inhibitory activity and binding energy suggest that these engagements occur primarily at the rim of the active pocket, rather than with the catalytic core. Conversely, compound **3**, while interacting with fewer residues, binds precisely to the core of the active site constituted by key residues such as THR-344, ASP-354, and ASP-357, forming a stronger and more direct hydrogen bond network [28]. This analysis underscores that the efficacy of a tyrosinase inhibitor is not merely a function of the number of interactions, but rather the strategic location and strength of those interactions within the enzyme’s active site.

### 2.6. Cell Viability Assay

#### 2.6.1. Effects on HaCaT Keratinocytes

**Figure 6:**
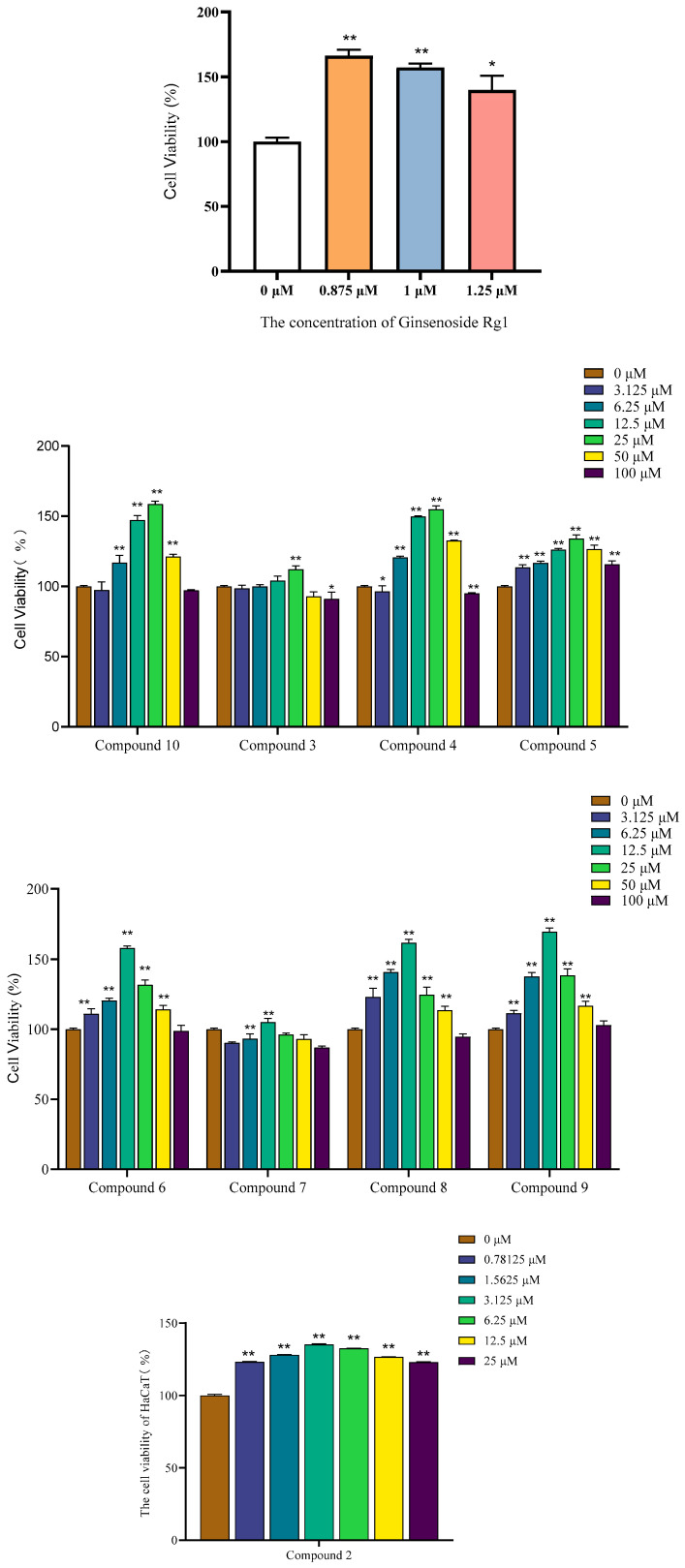
The cell viability of HaCaT cells with compounds. (*n* = 3; mean ± SD; * *p* < 0.05 and ** *p* < 0.01 vs. untreated control).

The promotion of keratinocyte proliferation is vital for skin barrier repair, wound healing, and maintaining skin integrity, which are key endpoints in anti-aging and regenerative dermatology [29]. HaCaT cells, immortalized human keratinocytes crucial for skin barrier function, were used to assess the proliferative effects of *C. oleifera* leaf compounds via CCK-8 assay. As shown in Table 4 and Figure 6, most compounds promoted HaCaT cell growth in a concentration-dependent manner, while high concentrations exerted cytotoxic effects. Ginsenoside Rg1 was used as a positive control for cell viability assays. Ginsenoside Rg1 (positive control) showed moderate proliferative effects (120–169% viability) at low concentrations (0.875–1.25 μM).

Compound **9** demonstrated the most potent proliferative effect, achieving a maximum viability of 169.30 ± 2.17% at 12.5 μM. Compound **8** also showed strong activity (161.72 ± 2.07% at 12.5 μM). The flavonoid compound **1** and polyphenol compound **4** and compound **6** also significantly enhanced proliferation, increasing viability by approximately 150% at their optimal concentrations. In contrast, compound **7** showed a negligible effect (104.97 ± 2.27% at 12.5 μM). Notably, all active compounds from *C. oleifera* leaves outperformed the positive control in promoting HaCaT proliferation. These findings confirm the potential of these compounds, especially specific flavonoids and polyphenols, to enhance keratinocyte proliferation.

Notably, all active *C. oleifera* compounds outperformed the positive control (Ginsenoside Rg1) in proliferative efficacy, particularly at mid-range concentrations (e.g., 12.5 μM). These results confirm that *C. oleifera* leaf compounds, particularly flavonoids and specific polyphenols, effectively promote HaCaT cell proliferation within optimal concentrations. The highest activity of Compound **9** suggests its potential as a key ingredient for skin regeneration applications. Overall, the findings highlight the therapeutic promise of these compounds in enhancing keratinocyte proliferation for cosmetic and dermatological uses.

The strong proliferative effect of compound **9**, a lignan glycoside, on keratinocytes is a novel and significant finding. While lignans are known for various biological activities, specific reports on their effects on HaCaT cell proliferation are scarce. This suggests a potential for this class of compounds in wound healing and skin regeneration applications. The strong activity of compound **1** is supported by a growing body of evidence. For instance, the protective effect of isoquercitrin on UVB-induced injury in cells and mouse skin [30].

#### 2.6.2. Effects on HFF-1 Fibroblasts

**Figure 7:**
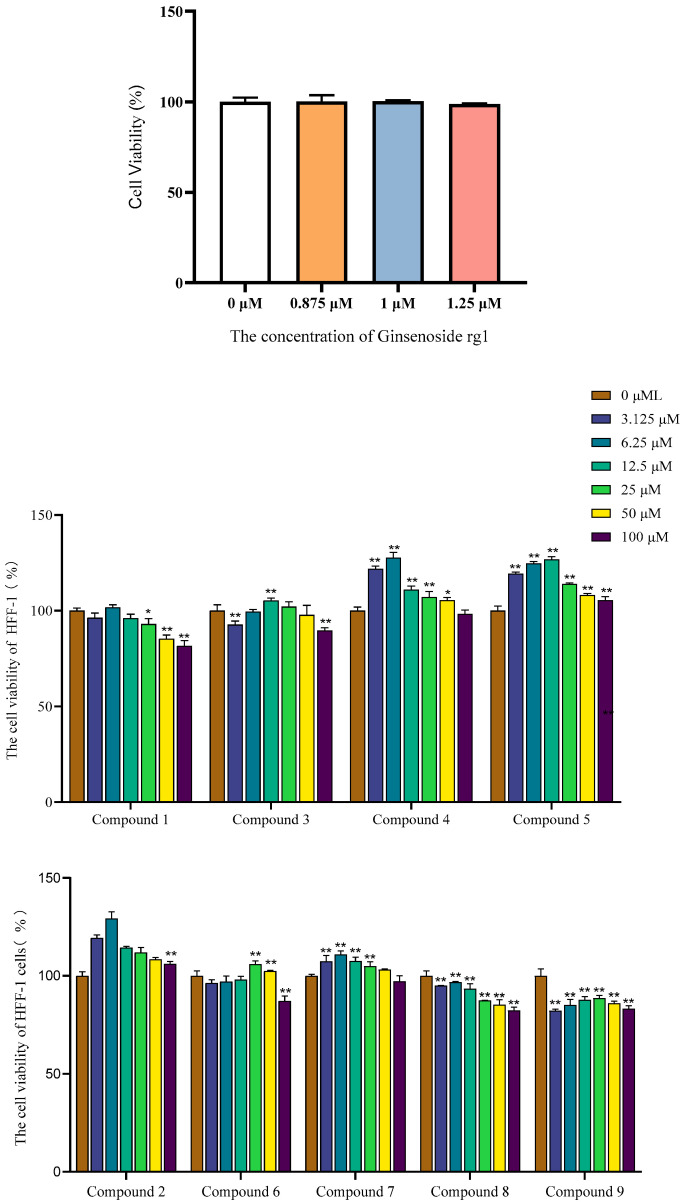
The cell viability of HFF-1 cells with compounds. (*n* = 3; mean ± SD; * *p* < 0.05 and ** *p* < 0.01 vs. untreated control).

Dermal fibroblasts are responsible for producing collagen and other extracellular matrix components. Stimulating their proliferation and activity is a fundamental strategy for improving skin elasticity, reducing wrinkles, and supporting skin rejuvenation [31,32,33]. The effects on human foreskin fibroblast (HFF-1) cells were also assessed (Table 5, Figure 7). The positive control, Ginsenoside Rg1, showed a mild proliferative effect.

The responses varied markedly among compounds. The flavonoid compound **3** exhibited the strongest proliferative effect, increasing HFF-1 viability to 129.36 ± 2.81% at 6.25 μM. The polyphenol compounds **4** and **5** also showed robust effects, with viability rates of 127.67 ± 2.14% and 126.16 ± 1.08% at their optimal concentrations, respectively. Conversely, compounds **8** and **9** demonstrated cytotoxic effects, reducing viability below 100% (96.72 ± 0.37% and 88.77 ± 1.17%, respectively). Other compounds induced only modest proliferation. These results indicate a differential impact of *C. oleifera* compounds on dermal fibroblasts, suggesting that their application in anti-aging cosmetics may require careful selection of specific compounds.

Furthermore, this study revealed that not all bioactive compounds promoted cell proliferation. Specifically, compound **8** and compound **9** exhibited significant cytotoxicity towards HFF-1 fibroblasts at the tested concentrations. While a detailed mechanistic investigation into this cytotoxicity (e.g., apoptosis induction, necrosis) falls outside the scope of this initial phytochemical and screening study, its identification is a critical safety consideration. The determination of cytotoxic thresholds provides essential data for the safe application of future extracts or formulations. The observed cytotoxicity warrants further investigation to elucidate the underlying mechanism, which is a primary focus of our ongoing research. Understanding whether this effect is mediated through apoptotic pathways, oxidative stress, or other mechanisms is imperative for a comprehensive risk-benefit assessment of these otherwise promising compounds. Quercetin effectively promoted cutaneous wound healing by enhancing the proliferation and migration of fibroblasts [34].

## 3. Materials and Methods

### 3.1. Chemicals

All chemicals were of analytical grade or higher. Ethanol, methanol, sodium nitrite (NaNO_2_), aluminum nitrate (Al(NO_3_)_3_), sodium hydroxide (NaOH), gallic acid, phloroglucinol, sodium carbonate (Na_2_CO_3_), L-tyrosine, mushroom tyrosinase, and kojic acid were purchased from Shanghai Maclin Biochemical Technology Co., Ltd (Shanghai, China). Phosphate-buffered saline (PBS), fluorescein sodium salt, 2,2′-azobis(2-methylpropionamidine) dihydrochloride (AAPH), L-ascorbic acid, 2,2-diphenyl-1-picrylhydrazyl (DPPH), Cell Counting Kit-8 (CCK-8), and Trolox were acquired from Titan Scientific Co., Ltd. (Shanghai, China). Cell culture components, including Dulbecco’s Modified Eagle Medium (DMEM), Minimum Essential Medium (MEM), fetal bovine serum (FBS), antibiotics, and trypsin-EDTA, were purchased from Gibco (Thermo Fisher Scientific, Inc. Waltham, MA, USA). HPLC-grade acetonitrile and methanol were obtained from Welch Materials Co., Ltd (Shanghai, China).

### 3.2. Plant Material and Extraction

*C. oleifera* leaves were collected in Liu’an City, Anhui Province, China, in March 2023. The plant material was identified by Professor Cunwu Chen, a botanist at the College of Biotechnology and Pharmaceutical Engineering, West Anhui University. A specimen (No. 20230423-2) has been deposited at the College of Biotechnology and Pharmaceutical Engineering (Room A211), West Anhui University.

Fresh leaves were dehydrated in a forced-air drying oven (Model DHG-9240A, Yiheng Scientific Instrument Co., Ltd., Shanghai, China) at 40 °C until brittle to facilitate grinding. The dried leaves were pulverized into a homogeneous powder using a laboratory grinder (Model TQ-500Y, Damai Machinery Co., Ltd., Yongkang, China).

For extraction, three parallel batches of powdered leaves (50 g each) were homogenized with 500 mL of solvent using a solid-to-solvent ratio of 1:10 (*w*/*v*). The solvents used were: ultrapure water (aqueous extract, WE), 50% ethanol (*v*/*v*) (hydroethanolic extract, HEE), and absolute ethanol (ethanolic extract, EE). Each suspension underwent ultrasonic-assisted extraction using an industrial sonicator (500 W, 40 kHz) for three 30 min cycles at 40 °C. The combined filtrates were clarified by centrifugation (8000× *g*, 5 min, 4 °C). The supernatant was concentrated under reduced pressure using a rotary evaporator and subsequently lyophilized (Freeze Dryer SCIENTZ-10N, Ningbo Scientz Biotechnology Co., Ltd., Ningbo, China) to obtain stable powdered extracts, labeled WE, HEE, and EE, respectively.

### 3.3. Isolation and Structure Elucidation of the Compounds from the C. oleifera Leaves Extracts

The HEE (200.0 g) was subjected to fractionation on an HP-20 macroporous resin column, eluted with a step gradient of ethanol in water (0%, 20%, 50%, 80%, 100% *v*/*v*). Five fractions (A–E) were collected. Fractions C (eluted with 50% ethanol, 32.85 g) and D (eluted with 80% ethanol, 19.72 g), which showed high bioactivity, were selected for further isolation.

Further purification was performed using semi-preparative HPLC on a Shimadzu system equipped with a C18 column (250 mm × 10 mm, 5 μm particle size). Elution was performed with a gradient of acetonitrile (A) and water (B) at a flow rate of 3 mL/min as follows: 0–40 min, 10–100% A; 40–50 min, 100% A. The column was subsequently re-equilibrated to the initial solvent composition, with detection at 254 nm. This process yielded nine pure compounds (**1**–**9**). Their structures were elucidated through comprehensive analysis of HR-ESI-MS, ^1^H-NMR, and ^13^C-NMR data, with comparisons to published literature (Detailed isolation procedures for each compound are provided in the Supplementary Materials).

### 3.4. Total Flavonoid Content (TFC) Assay

The TFC was determined using the aluminum chloride colorimetric method. A rutin standard solution (0.024 mg/mL) was used to generate a calibration curve. Briefly, sample or standard (0.5 mL) was mixed with 0.15 mL of 5% NaNO_2_. After 6 min, 0.15 mL of 10% AlCl_3_ was added, followed by 2 mL of 4% NaOH after another 6 min. The volume was adjusted to 5 mL with methanol, and the absorbance was measured at 510 nm after 15 min. The TFC was expressed as mg rutin equivalents per gram of dry extract (mg RE/g extract). All measurements were performed in triplicate.

### 3.5. Total Phenolic Content (TPC) Assay

The TPC was determined using the Folin–Ciocalteu method. A gallic acid standard solution (0.026 mg/mL) was used for calibration. Sample or standard (0.5 mL) was mixed with 2.5 mL of 10-fold diluted Folin–Ciocalteu reagent. After 4 min, 2 mL of 7.5% Na_2_CO_3_ solution was added. The mixture was incubated in the dark for 2 h at room temperature, and the absorbance was measured at 765 nm. The TPC was expressed as mg gallic acid equivalents per gram of dry extract (mg GAE/g extract). All measurements were performed in triplicate.

### 3.6. Bioactivity Assays

#### 3.6.1. DPPH Radical Scavenging Activity

The assay was performed in a 96-well plate. A sample solution (100 μL, at concentrations ranging from 10 to 500 μM in water) was mixed with 100 μL of a 0.06% (*w*/*v*) methanolic solution of DPPH. The plate was incubated in the dark at 37 °C for 30 min, and the absorbance was measured at 517 nm. L-Ascorbic acid (10–100 μM) was used as a positive control. The radical scavenging activity (RSA) was calculated as follows:

$$RSA(\%)=\frac{\left(A_{Control}-A_{Sample}\right)\times 100}{A_{Control}}$$

where A_control_ is the absorbance of the DPPH solution with solvent, and A_sample_ is the absorbance of the DPPH solution with the sample. The IC_50_ values were determined from the dose–response curves. Tests were conducted in triplicate.

#### 3.6.2. Tyrosinase Inhibition Assay

The assay was conducted in 0.1 M phosphate buffer (pH 6.8). The reaction mixture in each well contained 40 μL of sample (10–500 μM in PBS), 40 μL of 1.5 mM L-tyrosine substrate, 20 μL of mushroom tyrosinase (500 U/mL in PBS), and 40 μL of PBS. After incubation at 37 °C for 30 min, the absorbance was measured at 475 nm. Kojic acid (10–100 μM) was used as a positive control. The tyrosinase inhibition rate was calculated as follows:

$$nhibition\;\%=(1-\frac{A_{Sample}-A_{Sample\text{-}blank}}{A_{Control}-A_{Control\text{-}blank}})\times 100$$

where A_sample_ is the absorbance with sample and enzyme, A_sample-blank_ is the absorbance with sample and PBS (no enzyme), A_control_ is the absorbance with buffer and enzyme, and A_control-blank_ is the absorbance with buffer and PBS. Tests were conducted in triplicate.

#### 3.6.3. Molecular Docking Protocol

To gain insights into the potential inhibitory mechanism of the compounds against tyrosinase, molecular docking studies were performed using AutoDock (version 1.5.7) [35]. The 3D structures of the ligands were built and energy-minimized using Chem3D Ultra 22.0. The crystal structure of Agaricus bisporus tyrosinase (PDB ID: 2Y9X) was downloaded from the RCSB Protein Data Bank (http://www.rcsb.org, accessed on 26 August 2025). All water molecules were removed, and polar hydrogen atoms were added. Using AutoDockTools (version 1.5.4), Gasteiger charges were assigned, and a charge of +2 was assigned to each copper ion in the active site. The grid box was centered on the catalytic copper ions with dimensions sufficient to encompass the entire active site. Docking simulations were carried out using the Lamarckian genetic algorithm with 100 runs for each ligand. The conformation with the lowest binding energy (most negative value) was selected for analysis of the binding mode. Molecular graphics visualizations were generated using the PyMOL molecular graphics system (version 3.1.6.1).

#### 3.6.4. Cell Viability Assay (CCK8)

HaCaT and HFF-1 cells were cultured in DMEM supplemented with 10% FBS and 1% penicillin-streptomycin at 37 °C in a 5% CO_2_ humidified incubator. Cells in the exponential growth phase were seeded into 96-well plates at a density of 1 × 10^4^ cells/well and incubated for 24 h. The medium was then replaced with fresh medium containing 2% FBS and various concentrations of the test compounds. After 48 h of incubation, 10% CCK-8 reagent was added to each well, and the plates were incubated for another 3 h. The absorbance was measured at 450 nm using a microplate reader. Cell viability was calculated as follows:

$$Viability\;(\%)=\frac{A_{Sample}-A_{blank}}{A_{Control}-A_{blank}}\times 100$$

where A_sample_ is the absorbance of the treated cells, A_control_ is the absorbance of untreated cells, and A_blank_ is the absorbance of the medium alone. All experiments were performed in triplicate.

### 3.7. Statistical Analysis

Data are presented as mean ± standard deviation (SD) of three independent experiments. Statistical analysis was performed using GraphPad Prism 9.0 and IBM SPSS Statistics 26. Differences between groups were analyzed by one-way analysis of variance (ANOVA) followed by Tukey’s post hoc test. A value of *p* < 0.05 and *p* < 0.01 was considered statistically significant.

## 4. Conclusions

This study demonstrates that *C. oleifera* leaves are a rich source of bioactive flavonoids and polyphenols. The extraction solvent significantly influenced the yield and bioactivity of the extracts, with 50% ethanol proving most effective for recovering antioxidants and proliferative compounds.

We successfully isolated and identified nine compounds from the leaves. Key findings on their bioactivity include:

1. The flavonoid compound **1** exhibited exceptional antioxidant activity, surpassing that of L-ascorbic acid.
2. The isolated compounds showed generally modest tyrosinase inhibitory activity, indicating this may not be their primary mechanism of action.
3. Several compounds, notably compound **9** and the flavonoid compound **2**, significantly promoted the proliferation of human skin cells (HaCaT keratinocytes and HFF-1 fibroblasts, respectively), highlighting their potential for wound healing and anti-aging applications.

This work provides a strong chemical and pharmacological foundation for the valorization of *C. oleifera* leaf waste. Future research will focus on scaling up the isolation of key active compounds, establishing quantitative HPLC methods for quality control, elucidating detailed mechanisms of action, and exploring synergistic effects in formulations for cosmetic and pharmaceutical development.

## Figures and Tables

**Table 1:** Total flavonoid (TF), total phenolic (TP) contents, calibration equations (CE), and coefficients of determination (R^2^) of the extracts. Data are presented as mean ± SD (*n* = 3), Significance level (** *p* < 0.01).

Samples	TF(mg RE/g Extract)	TP (mg GAE/g Extract)
WE	120.52 ± 0.62 **	146.16 ± 1.89 **
HEE	189.23 ± 1.12 **	337.24 ± 1.94 **
EE	160.60 ± 1.08 **	160.58 ± 0.78 **

**Table 2:** Secondary metabolite of *Camellia oleifera* C. Abel leaves extracts.

Categories	No.	Compounds	Retention Time (min)
flavonoids	**1**	2-(3,4-Dihydroxyphenyl)-5,7-dihydroxy-4-oxo-4H-chromen-3-yl-glucopyranoside	24.783
	**2**	2-(3,4-Dihydroxyphenyl)-5,7-dihydroxy-4-oxo-4H-chromen-3-yl-6-deoxy-mannopyranoside	27.578
	**3**	2-(3,4-Dihydroxyphenyl)-5,7-dihydroxy-4-oxo-4H-chromen-3-yl-6-O-(6-Deoxy-mannopyranosyl)-glucopyranose	25.901
polyphenols	**4**	5-[2-(4-Hydroxyphenyl)ethyl]-1,3-benzenediol	28.202
	**5**	3-Hydroxy-5-[2-(4-hydroxyphenyl)ethyl]phenyl-glucopyranoside	25.966
	**6**	4-[2-(3,5-Dimethoxyphenyl)ethyl]phenyl-6-O-Arabinopyranosyl-glucopyranose	38.531
	**7**	4-[2-(3-Hydroxy-5-methoxyphenyl)ethyl]phenyl--6-O-(6-Deoxy-mannopyranosyl)-glucopyranose	39.439
	**8**	4-[2-(3,5-Dihydroxyphenyl)ethyl]phenyl-glucopyranoside	30.501
	**9**	[(1S,2R,3R)-7-Hydroxy-1-(4-hydroxy-3-methoxyphenyl)-3-(hydroxymethyl)-6-methoxy-1,2,3,4-tetrahydro-2-naphthalenyl]methyl β-D-xylopyranoside	23.501

**Table 3:** Tyrosinase inhibition rate of *Camellia oleifera* C. Abel l leaf compounds (%).

Compound	Inhibition Rate(%)
Kojic acid	77.76 ± 4.81
**1**	21.35 ± 2.19
**2**	25.18 ± 2.35
**3**	40.75 ± 2.73
**4**	37.99 ± 2.83
**5**	29.99 ± 2.27
**6**	31.89 ± 1.79
**7**	20.52 ± 1.07
**8**	26.64 ± 2.14
**9**	21.40 ± 1.63

**Table 4:** Proliferative effects of isolated compounds on HaCaT cells after 48 h treatment. Data represent percent viability relative to untreated control (mean ± SD, *n* = 3) at the most effective concentration for each compound.

Compound	Tested Concentration (μM)	Viability (%)
Ginsenoside Rg1	0.875	169.54 ± 1.52
**1**	25	158.74 ± 1.57
**2**	3.125	135.21 ± 0.37
**3**	25	112.36 ± 1.85
**4**	25	155.05 ± 1.93
**5**	25	134.09 ± 2.10
**6**	12.5	157.87 ± 1.27
**7**	12.5	104.97 ± 2.27
**8**	12.5	161.72 ± 2.07
**9**	12.5	169.30 ± 2.17

**Table 5:** Effects of isolated compounds on HFF-1 cell viability after 48 h treatment. Data represent percent viability relative to untreated control (mean ± SD, *5* = 3) at the tested concentration.

Compound	Tested Concentration (μM)	Viability (%)
**1**	6.25	101.74 ± 1.04
**2**	6.25	129.36 ± 2.81
**3**	12.5	105.25 ± 1.03
**4**	6.25	127.67 ± 2.14
**5**	12.5	126.16 ± 1.08
**6**	25	105.98 ± 1.42
**7**	6.25	110.94 ± 1.43
**8**	6.25	96.72 ± 0.37
**9**	25	88.77 ± 1.17

## Data Availability

The data presented in this study are available in the article and Supplementary Material.

## Supplementary Materials

The following supporting information can be downloaded at: https://www.mdpi.com/article/10.3390/molecules30183661/s1. Detailed HR-ESI-MS and NMR spectra (^1^H, ^13^C) for all isolated compounds (**1**–**9**).
