# Supplementary material for: Chemical Composition and Bioactive Properties of Camellia oleifera C. Abel Leaves
Source: Molecules. 2025 Sep 9;30(18):3661. doi: 10.3390/molecules30183661 (PMC12472427; doi:10.3390/molecules30183661)
Supplement: Supplementary file 1 [file molecules-30-03661-s001.zip › molecules-3736217-supplementary.pdf]

Supplementary Materials

Chemical Composition and Bioactive Properties of *Camellia oleifera* C. Abel Leaves

Jun Chen[1,†], Lilin Xiang[1,†], Deliang Qiao[1], Changli Min[1], Li Zhang[2], Xuejun Wang[1,*]

[1] College of Biotechnology and Pharmaceutical Engineering, West Anhui University, Lu'an 237012, China; 02000179@wxc.edu.cn (J.C.); lilin.xiang@rainbowfish11000.com (L.X.); qiaodl@wxc.edu.cn (D.Q.); mcl0917@163.com (C.M.)

[2] Analytical and Testing Center, West Anhui University, Lu'an 237012, China; 02000081@wxc.edu.cn

* Correspondence: 02000054@wxc.edu.cn

† These authors contributed equally to this work.

Methods

Isolation and Structure Elucidation of the Compounds from the *C. oleifera* C. Abel leaves extracts

Figure S1. ^{1}H NMR spectrum (850 MHz) data of compound **1** in DMSO-d6
Figure S2. ^{13}C NMR spectrum (210 MHz) data of compound **1** in DMSO-d6
Figure S3. HRESIMS spectrum of compound **1**
Figure S4. ^{1}H NMR spectrum (850 MHz) data of compound **2** in DMSO-d6
Figure S5. ^{13}C NMR spectrum (210 MHz) data of compound **2** in DMSO-d6
Figure S6. HRESIMS spectrum of compound **2**
Figure S7. ^{1}H NMR spectrum (850 MHz) data of compound **3** in DMSO-d6
Figure S8. ^{13}C NMR spectrum (210 MHz) data of compound **3** in DMSO-d6
Figure S9. HRESIMS spectrum of compound **3**
Figure S10. ^{1}H NMR spectrum (850 MHz) data of compound **4** in DMSO-d6
Figure S11. ^{13}C NMR spectrum (210 MHz) data of compound **4** in DMSO-d6
Figure S12. HRESIMS spectrum of compound **4**
Figure S13. ^{1}H NMR spectrum (850 MHz) data of compound **5** in DMSO-d6
Figure S14. ^{13}C NMR spectrum (210 MHz) data of compound **5** in DMSO-d6
Figure S15. HRESIMS spectrum of compound **5**
Figure S16. ^{1}H NMR spectrum (850 MHz) data of compound **6** in DMSO-d6
Figure S17. ^{13}C NMR spectrum (210 MHz) data of compound **6** in DMSO-d6
Figure S18. HRESIMS spectrum of compound **6**
Figure S19. ^{1}H NMR spectrum (850 MHz) data of compound **7** in DMSO-d6
Figure S20. ^{13}C NMR spectrum (210 MHz) data of compound **7** in DMSO-d6
Figure S21. HRESIMS spectrum of compound **7**
Figure S22. ^{1}H NMR spectrum (850 MHz) data of compound **8** in DMSO-d6
Figure S23. ^{13}C NMR spectrum (210 MHz) data of compound **8** in DMSO-d6
Figure S24. HRESIMS spectrum of compound **8**
Figure S25. ^{1}H NMR spectrum (850 MHz) data of compound **9** in DMSO-d6
Figure S26. ^{13}C NMR spectrum (210 MHz) data of compound **9** in DMSO-d6
Figure S27. HRESIMS spectrum of compound **9**
Reference

1. Methods

1.1. Isolation of the Compounds from the *C. oleifera* Abel leaves ext*racts*

C. oleifera Abel leaves extracts were subjected to HP-20 macroporous resin column chromatography, and was eluted with different ratios of ethanol-water (0, 20, 50, 80, 100% ethanol). The eluates were collected to obtain five components: A (eluted with water), B (eluted with 20% ethanol), C (eluted with 50% ethanol), D (eluted with 80% ethanol), and E (eluted with 100% ethanol). Components C (32.85 g) and D (19.72 g) were selected for further isolation of active chemical constituents. Component C and D were purified using a SHIMADZU C18 column (250 mm × 10 mm, 5 μm particle size) to afford 10 compounds. The structures of these compounds were elucidated by NMR and MS.

1.1.1 Compounds 1-3

Compounds 2 (5.4 mg, tR = 24.783 min) were isolated from fraction C-5 by semi-preparative HPLC with 20% acetonitrile elution (flow rate: 3 mL/min, 254 nm detection). Compound 3 (12.7 mg, tR = 27.578 min) was obtained from fraction D-1 under the same chromatographic conditions, and Compound 4 (16.8 mg, tR = 25.901 min) was isolated from fraction C-7.

1.1.2. Compounds 3-9

Compounds 3–9 were isolated using semi-preparative HPLC with varying acetonitrile concentrations: Compound 4 (8.6 mg, tR = 28.202 min) from fraction D-7 (22.5% acetonitrile); Compounds 5 (62.7 mg, tR = 25.966 min) and 6 (546.7 mg, tR = 38.531 min) from fraction D-8 (30% acetonitrile); Compound 7 (7.7 mg, tR = 39.439 min) from fraction C-5 (20% acetonitrile); Compound 8 (7.9 mg, tR = 30.501 min) from fraction C-3 (20% acetonitrile); Compound 9 (5.1 mg, tR = 23.501 min) from fraction C-7 (20% acetonitrile).

1.2. Structure Elucidation of the Compounds from the *C. oleifera* Abel leaves ext*racts*

1.2.1. Compound 1

Yellow powder was examined by HR-ESIMS, showing a molecular ion peak at m/z 487.0863 $[M+Na]^+$ (calcd 487.0852), with a molecular formula is $C_{21}H_{20}O_{12}$. Analyzed by ^{1}H-NMR (850 MHz, DMSO-d_6) δ: 7. 06 (d, J = 2.0 Hz, 1H, H-2′), 6.84 (d, J = 8.5 Hz, 1H, H-5′), 6.16 (d, J = 1.8 Hz, 1H, H-6), 5.45 (d, J = 7.7 Hz, 1H, H-1″), 4.88 (d, J = 5.3 Hz, 1H, OH-2″), 3.82 (dd, J = 11.8, 5.0 Hz, 1H, OH-3″), 3.74 (m, 1H, H-5″), 3.58 (m, 2H, H-6″). ^{13}C-NMR (210 MHz, DMSO-d_6) δ: 177.8 (C-4), 166.4 (C-7), 161.7 (C-5), 156.5 (C-2,9), 146.5 (C-4′), 145.3 (C-3′), 133.7 (C-3), 122.1 (C-1′), 121.5 (C-6′), 116.6 (C-5′), 115.7 (C-2′), 109.6 (C-10), 104.1 (C-1″), 99.4 (C-6), 94.1 (C-8), 77.0 (C-5″), 73.0 (C-3″), 70.4 (C-2″), 61.4 (C-4″). Consistent with the data presented in the literature [1]. Identify it as 2-(3,4-Dihydroxyphenyl)-5,7-dihydroxy-4-oxo-4H-chromen-3-yl-glucopyranoside.

1.2.2. Compound 2

Yellow powder was examined by HR-ESIMS, showing a molecular ion peak at m/z 471.0905 $[M+Na]^+$ (calcd 471.0903), with a molecular formula is $C_{21}H_{20}O_{11}$. Analyzed by ^{1}H-NMR (850 MHz, DMSO-d_6) δ: 7.25 (m, 1H, H-6′), 6.87 (dd, J = 17.8, 8.5 Hz, 1H, H-5′), 6.40 (dd, J = 8.8, 2.0 Hz, 1H, H-8), 6.21 (t, J = 2.1 Hz, 1H, H-6), 5.26 (dd, J = 15.3, 1.2 H z, 1H, H-1″), 3.98 (s, 1H, H-2″), 0.88 (dd, J = 10.1, 5.9 Hz, 3H,H-6″). ^{13}C-NMR (210 MHz, DMSO-d_6) δ: 178.2 (C-4), 164.7 (C-7), 161.8 (C-5), 160.0 (C-6′), 157.8 (C-9), 156.9 (C-2), 148.9 (C-4′), 145.9 (C-3′), 134.7 (C-3), 121.6 (C-5′), 121.2 (C-1′), 115.9 (C-2′), 104.5 (C-10), 101.8 (C-1″), 99.1 (C-6), 94.1 (C-8), 71.1 (C-2″), 70.6 (C-4″), 70.3 (C-5″), 70.1 (C- 3″), 18.0 (-CH3). Consistent with the data presented in the literature [2]. Identify it as 2-(3,4-Dihydroxyphenyl)-5,7-dihydroxy-4-oxo-4H-chromen-3-yl-6-deoxy-mannopyranoside.

1.2.3. Compound 3

White powder was examined by HR-ESIMS, showing a molecular ion peak at m/z 617.1501 $[M+Na]^+$ (calcd 617.1482)，with a molecular formula is $C_{27}H_{30}O_{15}$. Analyzed by ^{1}H-NMR (850 MHz, DMSO-d_6) δ: 6.88 (d, 8.8 Hz, 2H, H-3′, H-5′), 6.4 (s, 1H, H-8), 6.19 (s, 1H, H-6), 5.36 (d, 7.3 Hz 1H, H-1″), 4.58 (1H, H-1‴), 3.78–3.28 (10 H). ^{13}C-NMR (210 MHz, DMSO-d6) δ: 177.8 (C-4), 166.4 (C-7), 161.60 (C-4′), 160.4 (C-5), 157.2 (C-9), 157.0 (C-2), 133.7 (C-3), 131.3 (C-2′, C-6′), 121.4 (C-1′), 115.6 (C-3′，C-5′), 104.3 (C-10), 102.4 (C-1″, C-1‴), 99.3 (C-6), 95.1 (C-8), 76.8 (C-3″), 76.2 (C-5″), 74.6 (C-2″), 73.8 (C-4‴), 73.7 (C-4″), 72.3 (C-2‴), 71.1 (C-3‴), 69.7 (C-5‴), 68.7 (C-6″), 18.2 (C-6‴). Consistent with the data presented in the literature [3]. Identify it as 2-(3,4-Dihydroxyphenyl)-5,7-dihydroxy-4-oxo-4H-chromen-3-yl-6-O-(6-Deoxy-mannopyranosyl)-glucopyranose.

1.2.4. Compound 4

White powder was examined by HR-ESIMS, showing a molecular ion peak at m/z 229.0861 $[M+H]^-$ (calcd 229.0841), with a molecular formula is $C_{14}H_{14}O_3$. Analyzed by ^{1}H-NMR (850 MHz, DMSO-d_6) δ: 7.00 (m, 8.5Hz, 1H, H-2″, H-6″), 6.99 (m, 8.5Hz, 1H, H-3″, H-5″), 6.65(d, 2.0 Hz, 1H, H-2′, H-6′), 6.30 (t, 2.0 Hz, 1H, H-4′), 2.68 (m, 4.0 Hz, 2H, H-1). ^{13}C-NMR (210 MHz, DMSO-d_6) δ: 158.6 (C-3′), 158.6 (C-4′), 155.8 (C-4″), 144.1 (C-1′), 132.2 (C-1″), 129.6 (C-2″), 129.6 (C-6″), 115.4 (C-3″), 115.4 (C-5″), 106.7 (C-2′), 106.7 (C-6′), 100.6 (C-4′), 38.1 (C-2), 36.5 (C-1). Consistent with the data presented in the literature [4]. Identify it as 5-[2-(4-Hydroxyphenyl)ethyl]-1,3-benzenediol.

1.2.5. Compound 5

White powder was examined by HR-ESIMS, showing a molecular ion peak at m/z 575.2104 $[M+Na]^+$ (calcd 575.2104), with a molecular formula is $C_{27}H_{36}O_{12}$. Analyzed by ^{1}H-NMR (850 MHz, DMSO-d_6) δ: 7.16 (d, 8.6Hz, 1H, H-2″, H-6″), 6.97 (d, 8.6Hz, 1H, H-3″, H-5″), 6.39 (d, 2.3 Hz, 1H, H-6′), 6.30 (d, 2.3 Hz, 1H, H-4′), 3.94 (s, 1H, H-3′, H-5′), 2.79 (t, 6.8Hz, 2H, H-1, H-2). ^{13}C-NMR (210 MHz, DMSO-d_6) δ: 160.8 (C-3′), 160.8 (C-5′), 156.1 (C-4″), 144.4 (C-1′), 135.3 (C-1″), 129.7 (C-2″), 129.7 (C-6″), 117.7 (C-5″), 116.7 (C-3″), 109.2 (C-4′), 109.2 (Glu-C-1), 109.2 (Rha-C-1), 106.9 (C-2′), 106.9 (C-6′), 77.0 (Glu-C-3), 76.3 (Glu-C-5), 74.9 (Glu-C-2), 73.5 (Rha-C-4), 73.0 (Rha-C-3), 71.8 (Glu-C-4), 71.8 (Rha-C-2), 70.3 (Rha-C-5), 67.8 (Glu-C-6), 38.1 (C-2), 36.5 (C-1), 17.9 (Rha-C-6). Consistent with the data presented in the literature [5]. Identify it as 3-Hydroxy-5-[2-(4-hydroxyphenyl)ethyl]phenyl-glucopyranoside.

1.2.6. Compound 6

White powder was examined by HR-ESIMS, showing a molecular ion peak at m/z 589.2261$[M+Na]^+$ (calcd 589.2261), with a molecular formula is $C_{28}H_{38}O_{12}$. Analyzed by ^{1}H-NMR (850 MHz, DMSO-d_6) δ: 6.94 (m, 8.5Hz, 1H, H-3″, H-5″), 6.4 (d, 2.0Hz, 1H, H-2′, H-6′), 7.17 (m, 8.5Hz, 1H, H-2″, H-6″), 6.31 (t, 2.0 Hz, 1H, H-4′), 3.42 (s, 1H, H-7′, H-8′), 2.81 (m, 4.0Hz, 2H, H-1, H-2). ^{13}C-NMR (210 MHz, DMSO-d_6) δ: 160.8 (C-3′), 160.8 (C-5′), 156.1 (C-4″), 144.4 (C-1′), 135.3 (C-1″), 129.7 (C-2″), 129.7 (C-6″), 116.7 (C-3″), 116.7 (C-5″), 109.2 (Glu-C-1), 109.2 (Rha-C-1), 106.9 (C-2′), 106.9 (C-6′), 98.2 (C-4′), 77.0 (Glu-C-3), 73.5 (Glu-C-2), 73.5 (Glu-C-5), 73.0 (Rha-C-4), 71.8 (Rha-C-3), 70.5 (Rha-C-2), 70.3 (Glu-C-4), 67.8 (Rha-C-5), 65.3 (Glu-C-6), 55.5 (C-7′), 55.5 (C-8′), 38.1 (C-1), 36.5 (C-2), 17.9 (Rha-C-6). Consistent with the data presented in the literature [5]. Identify it as 4-[2-(3,5-Dimethoxyphenyl)ethyl]phenyl-6-O-Arabinopyranosyl-glucopyranose.

1.2.7. Compound 7

White powder was examined by HR-ESIMS, showing a molecular ion peak at m/z 415.1367 $[M+Na]^+$ (calcd 415.1369), with a molecular formula is $C_{20}H_{34}O_8$. Analyzed by ^{1}H-NMR (850 MHz, DMSO-d_6) δ: 6.92 (d, 8.0Hz, 1H, H-2″, H-6″), 6.30 (s, 1H, H-2′, H-6′), 6.07 (s, 1H, H-4′), 2.76 (t, 6.8Hz, 2H, H-1, H-2). ^{13}C-NMR (210 MHz, DMSO-d_6) δ: 158.6 (C-3′), 158.6 (C-5′), 156.1 (C-4″), 143.9 (C-1′), 135.4 (C-1″), 129.6 (C-2″), 129.6 (C-6″), 116.5 (C-5″), 116.5 (C-3″), 109.2 (Glu-C-1), 106.9 (C-6′), 100.6 (C-4′), 77.5 (Glu-C-3), 77.1 (Glu-C-5), 73.7 (Glc-C-2), 70.2 (Glu-C-4), 61.2 (Glu-C-6), 37.9 (C-1), 36.4 (C-2). Consistent with the data presented in the literature [5]. Identify it as 4-[2-(3-Hydroxy-5-methoxyphenyl)ethyl]phenyl--6-O-(6-Deoxy-mannopyranosyl)-glucopyranose.

1.2.8. Compound 8

White powder was examined by HR-ESIMS, showing a molecular ion peak at m/z 415.1372 $[M+Na]^+$ (calcd 415.1369), with a molecular formula is $C_{20}H_{24}O_8$. Analyzed by ^{1}H-NMR (850 MHz, DMSO-d_6) δ: 7.00 (d, 1.8 Hz, 1H, H-2, H-6), 6.65 (d, 1.8 Hz, 1H, H-3, H-5), 6.44 (d, 1.8 Hz, 1H, H-10), 6.28 (s, 3H, H-14), 4.56 (d, 5.7 Hz, 1H, H-7), 2.50 (d, 12.5 Hz, 1H, H-8). ^{13}C-NMR (210 MHz, DMSO-d_6) δ: 160.0 (C-11), 158.7 (C-4), 156.1 (C-13), 144.2 (C-9), 135.5 (C-1), 129.7 (C-2), 129.6 (C-6), 116.7 (C-3), 115.4 (C-5), 108.4 (C-14), 105.4 (Glc-C-1), 101.3 (C-12), 100.9 (C-10), 77.5 (Glc-C-5), 77.1 (Glc-C-3), 73.7 (Glc-C-2), 70.1 (Glc-C-4), 61.1 (Glc-C-6), 38.0 (C-7), 36.4 (C-8). onsistent with the data presented in the literature [6]. Identify it as 4-[2-(3,5-Dihydroxyphenyl)ethyl]phenyl-glucopyranoside.

1.2.9. Compound 9

White powder was examined by HR-ESIMS, showing a molecular ion peak at m/z 253.0861 $[M+Na]^+$ (calcd 515.1893)， with a molecular formula is $C_{25}H_{32}O_{10}$. Analyzed by ^{1}H-NMR (850 MHz, DMSO-d_6) δ:6.70 (s, 1H, H-5), 6.69 (d, 1.8 Hz, 1H, H-6'), 6.61 (s, 1H, H-2), 4.03 (s, 3H, OCH3), 3.93 (s, 3H, OCH3), 2.93 (m, 2H, H-7), 1.70 (m, 2H, H-8, 8'). ^{13}C-NMR (210 MHz, DMSO-d_6) δ: 149.8 (C-3'), 147.7(C-3), 144.6 (C-4'), 144.0 (C-4), 136.5 (C-1'), 132.6 (C-6), 129.3 (C-1), 121.7 (C-6'), 118.3 (C-5'), 115.8 (C-5), 113.8 (C-2'), 111.2 (C-2), 110.9 (C-1″), 77.0 (C-3″), 73.7 (C-2″), 69.5 (C-4″), 68.1 (C-9'), 66.2 (C-5″), 63.6 (C-9), 56.0 (OCH3), 56.0 (-OCH3), 46.7 (C-7'), 44.1 (C-8'), 39.0 (C-8), 32.7 (C-7). Consistent with the data presented in the literature [7]. Identify it as [(1S,2R,3R)-7-Hydroxy-1-(4-hydroxy-3-methoxyphenyl)-3-(hydroxymethyl)-6-methoxy-1,2,3,4-tetrahydro-2-naphthalenyl]methyl β-D-xylopyranoside.

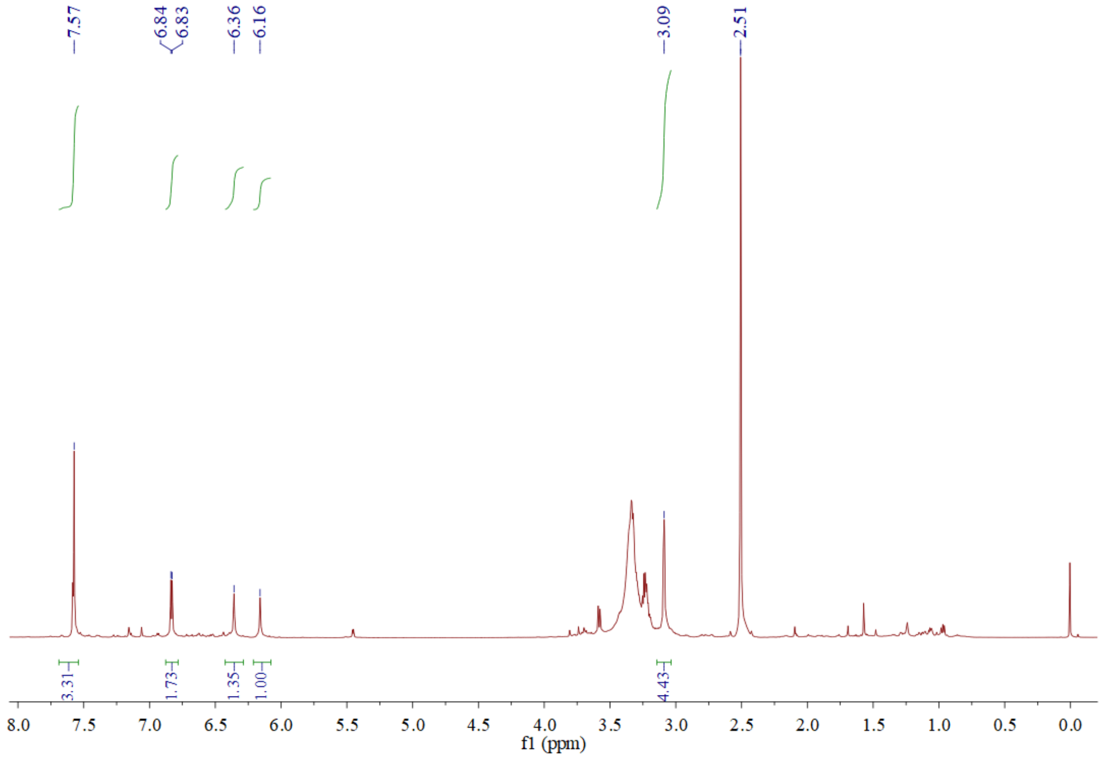

Figure S1. ^{1}H NMR spectrum (850 MHz) data of compound **1** in DMSO-d6

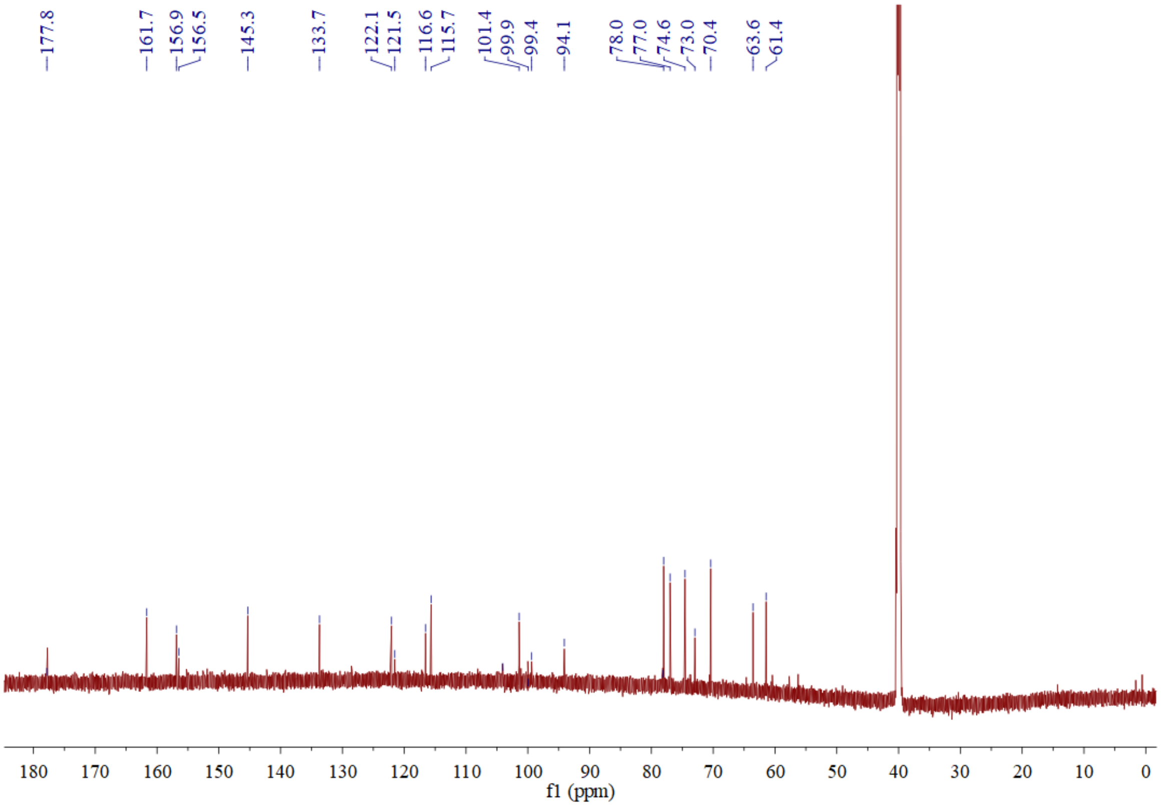

Figure S2. ^{13}C NMR spectrum (210 MHz) data of compound **1** in DMSO-d6

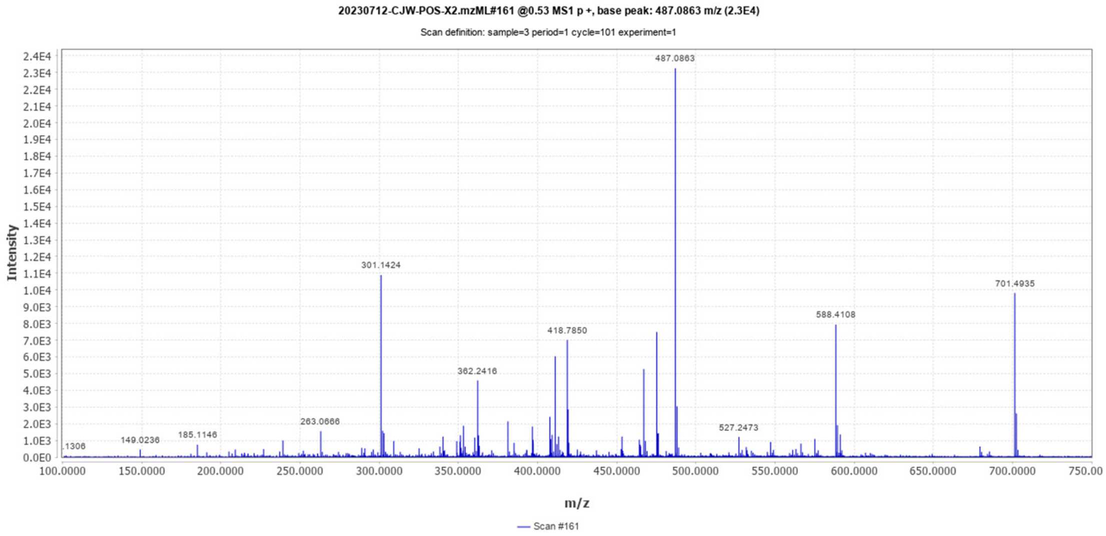

Figure S3. HRESIMS spectrum of compound **1**

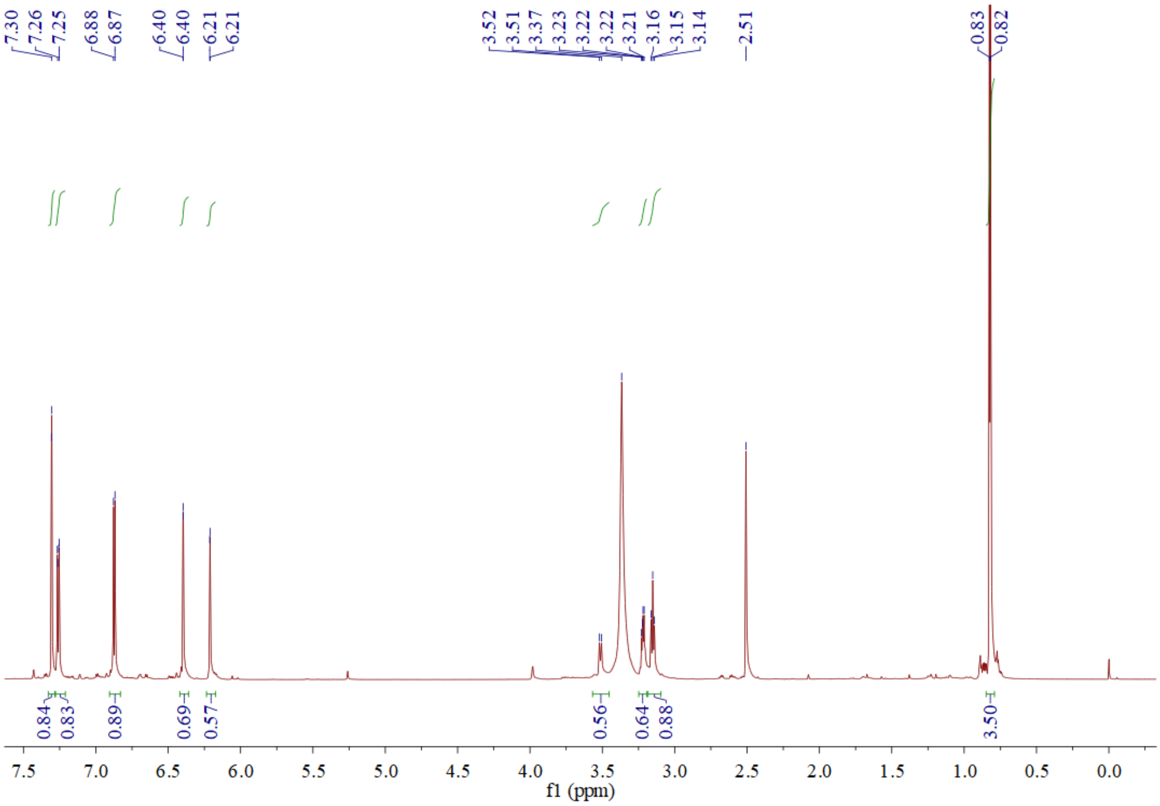

Figure S4. ^{1}H NMR spectrum (850 MHz) data of compound **2** in DMSO-d6

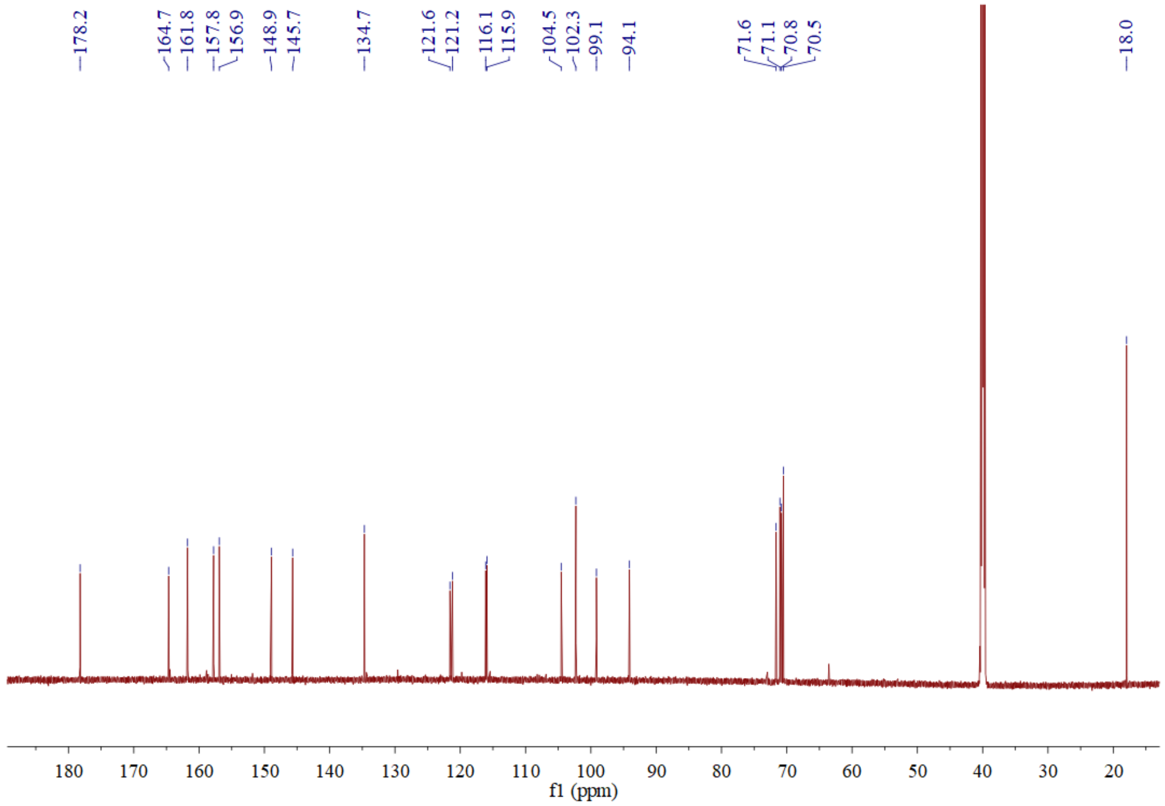

Figure S5. ^{13}C NMR spectrum (210 MHz) data of compound **2** in DMSO-d6

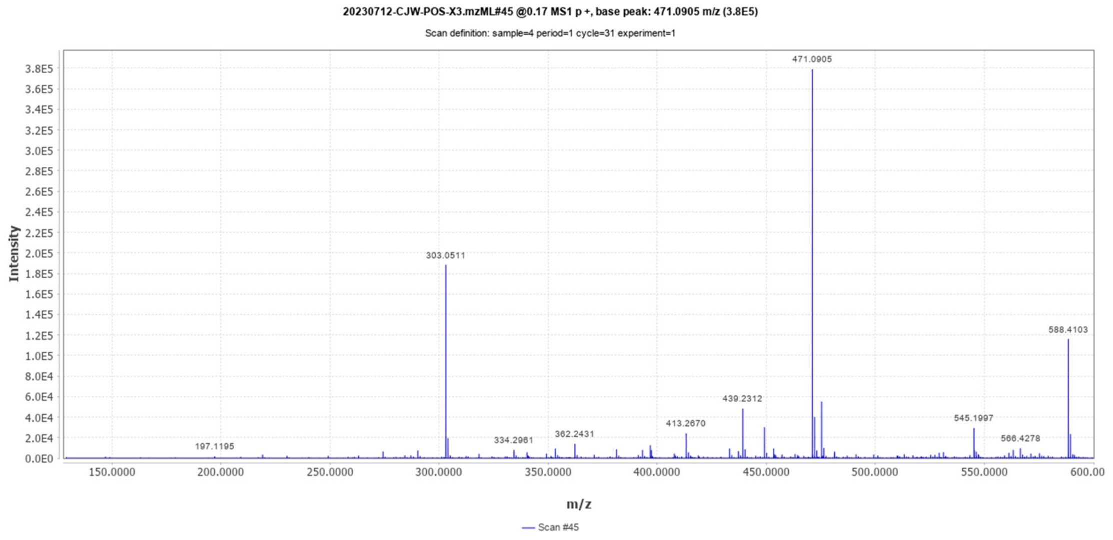

Figure S6. HRESIMS spectrum of compound **2**

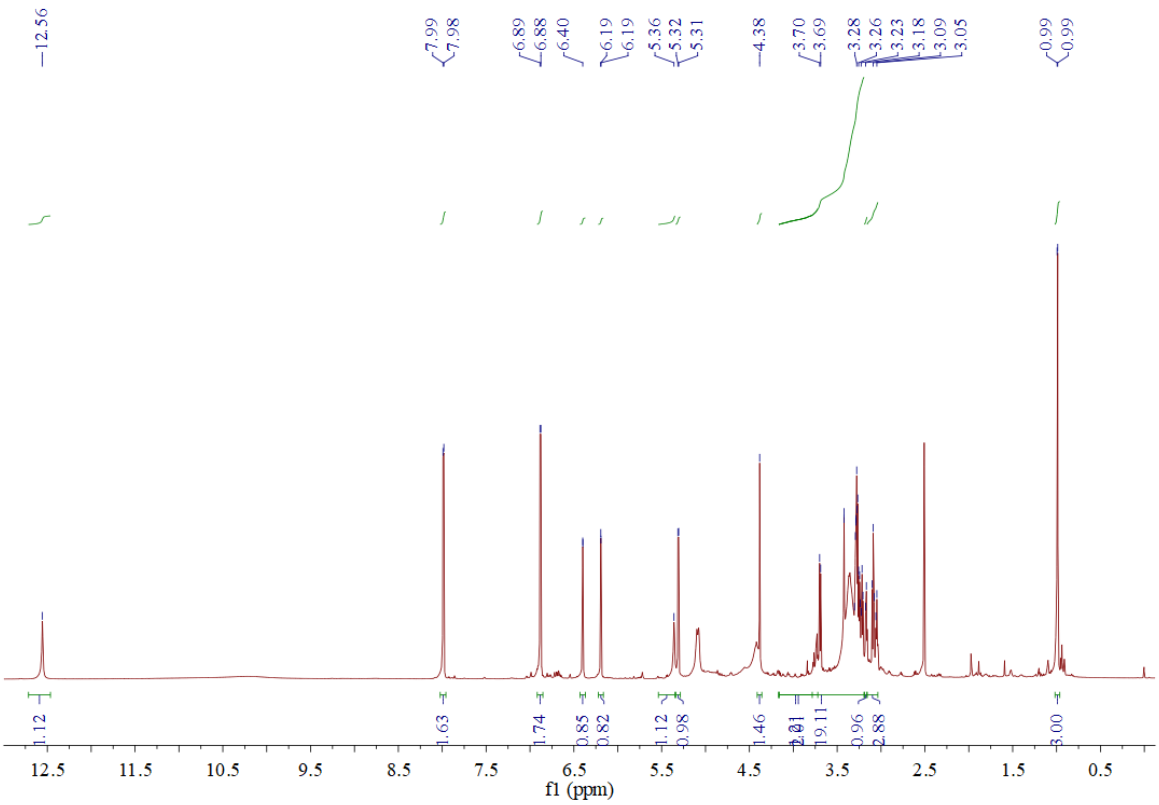

Figure S7. ^{1}H NMR spectrum (850 MHz) data of compound **3** in DMSO-d6

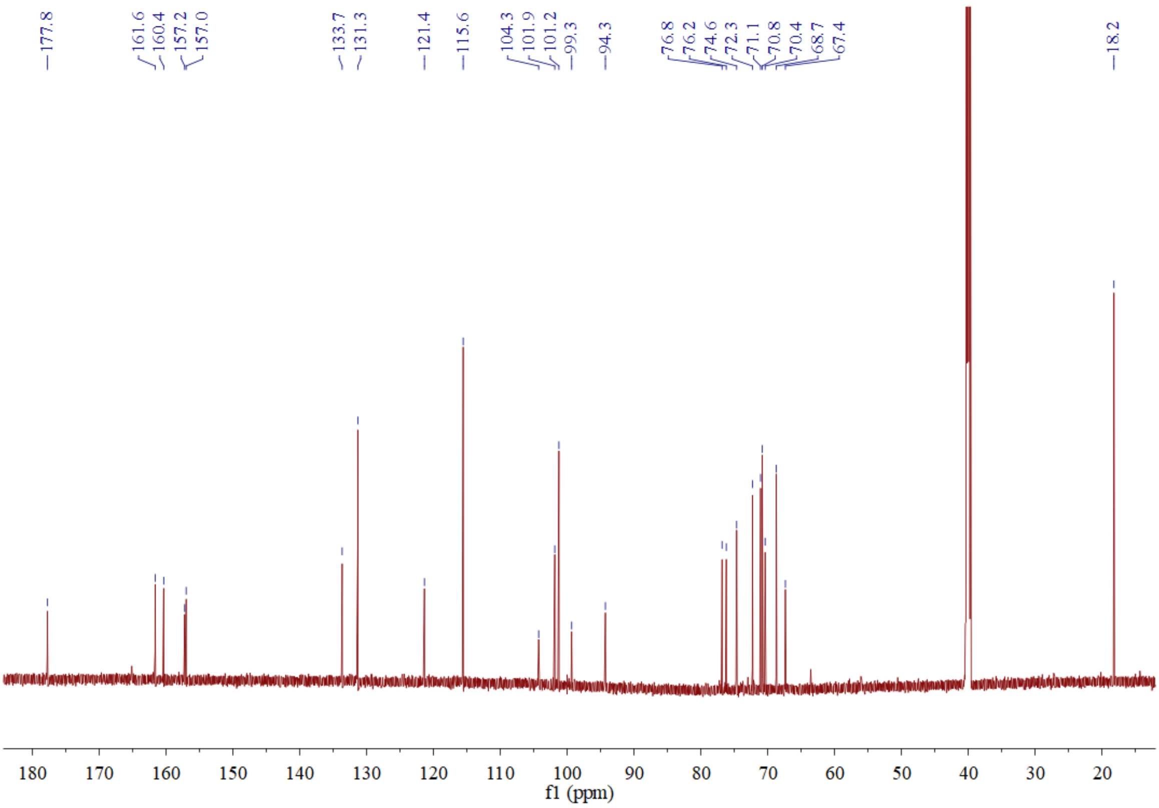

Figure S8. ^{13}C NMR spectrum (210 MHz) data of compound **3** in DMSO-d6

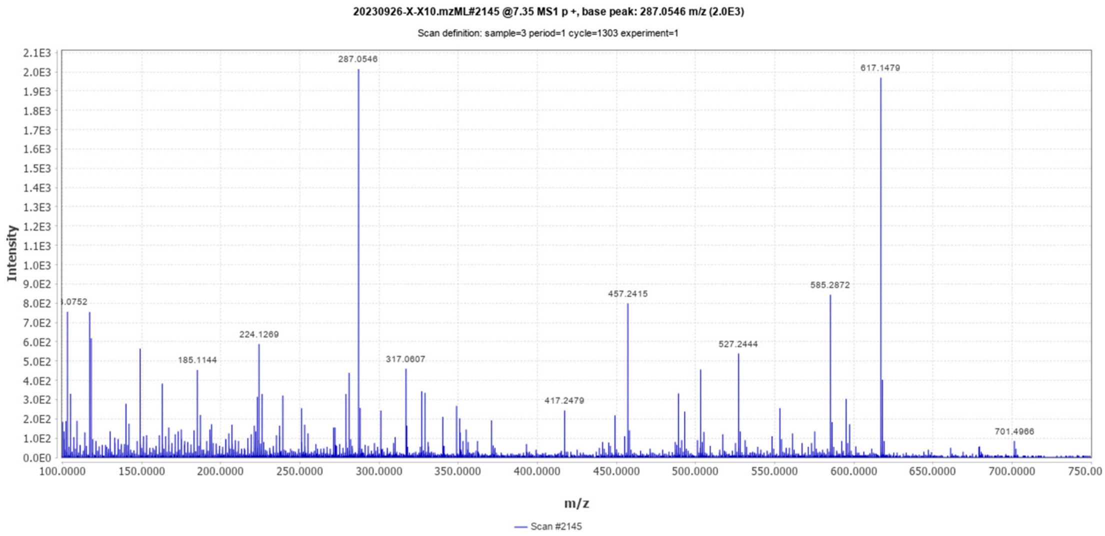

Figure S9. HRESIMS spectrum of compound **3**

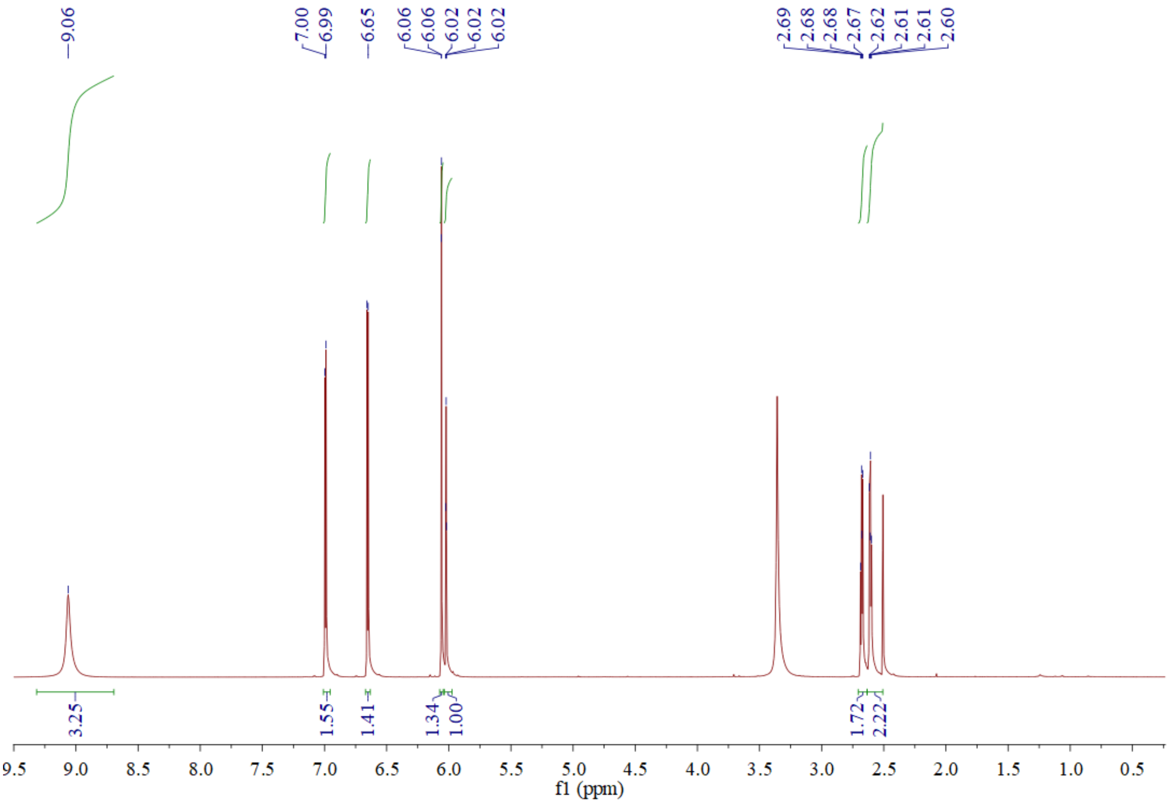

Figure S10. ^{1}H NMR spectrum (850 MHz) data of compound **4** in DMSO-d6

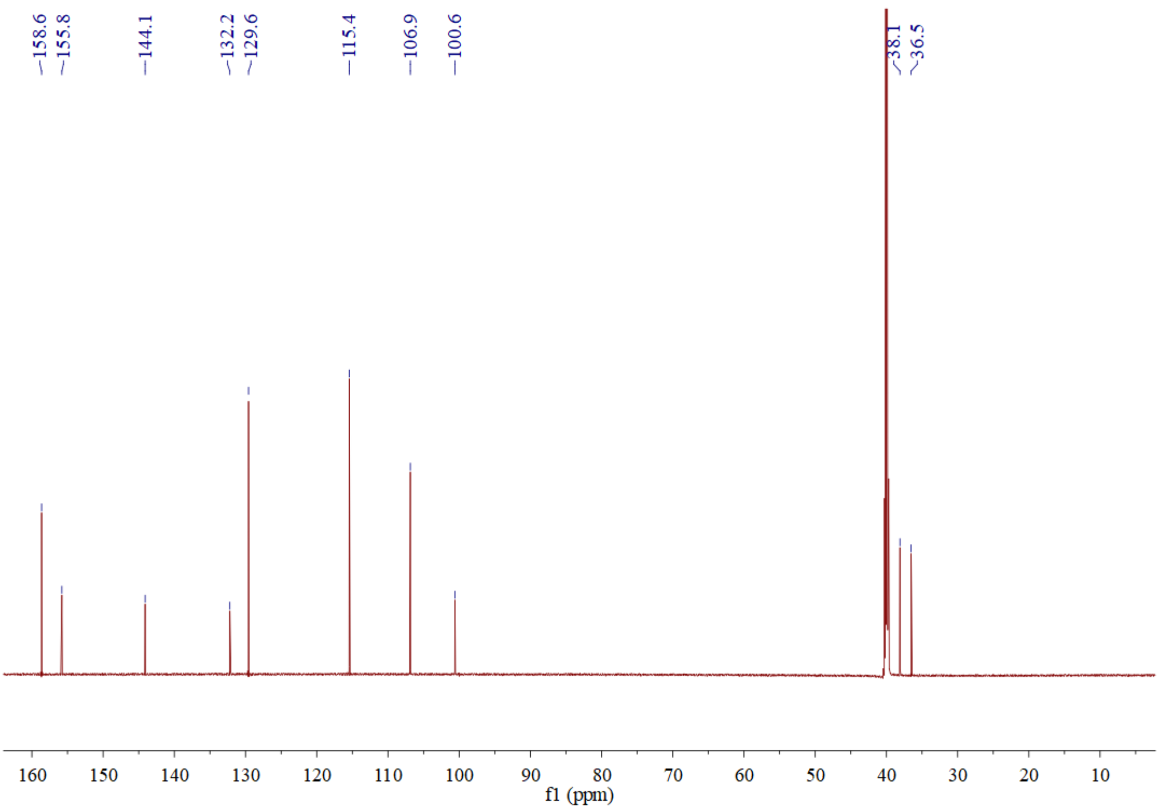

Figure S11. ^{13}C NMR spectrum (210 MHz) data of compound **4** in DMSO-d6

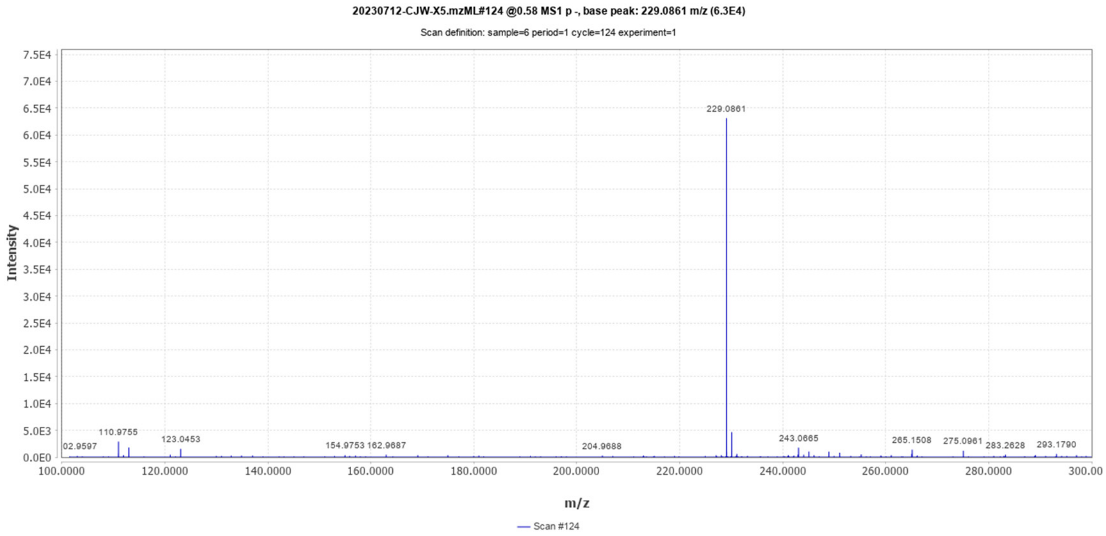

Figure S12. HRESIMS spectrum of compound **4**

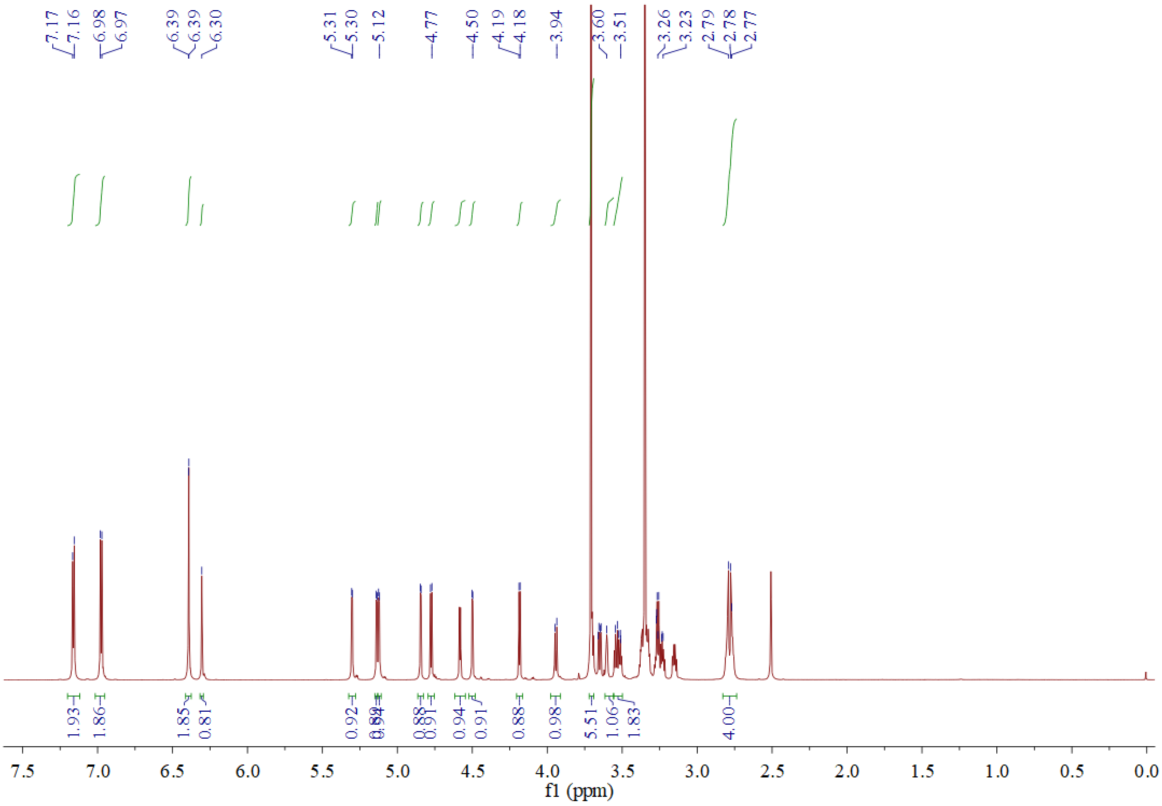

Figure S13. ^{1}H NMR spectrum (850 MHz) data of compound **5** in DMSO-d6

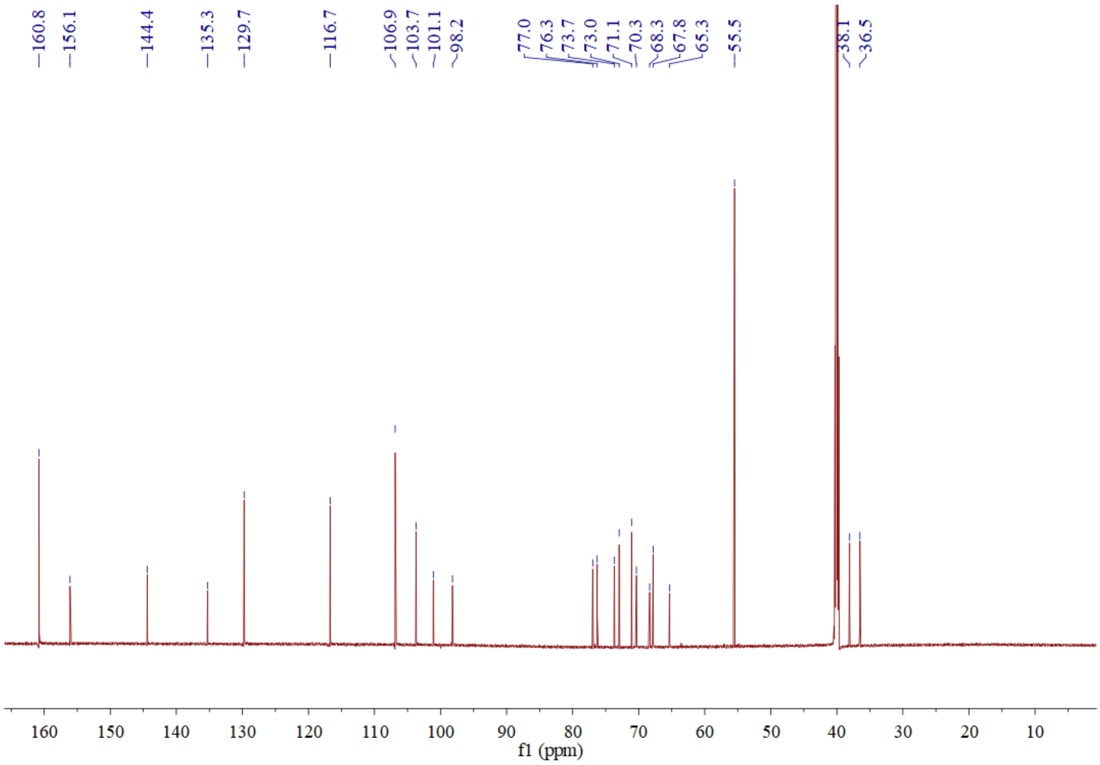

Figure S14. ^{13}C NMR spectrum (210 MHz) data of compound **5** in DMSO-d6

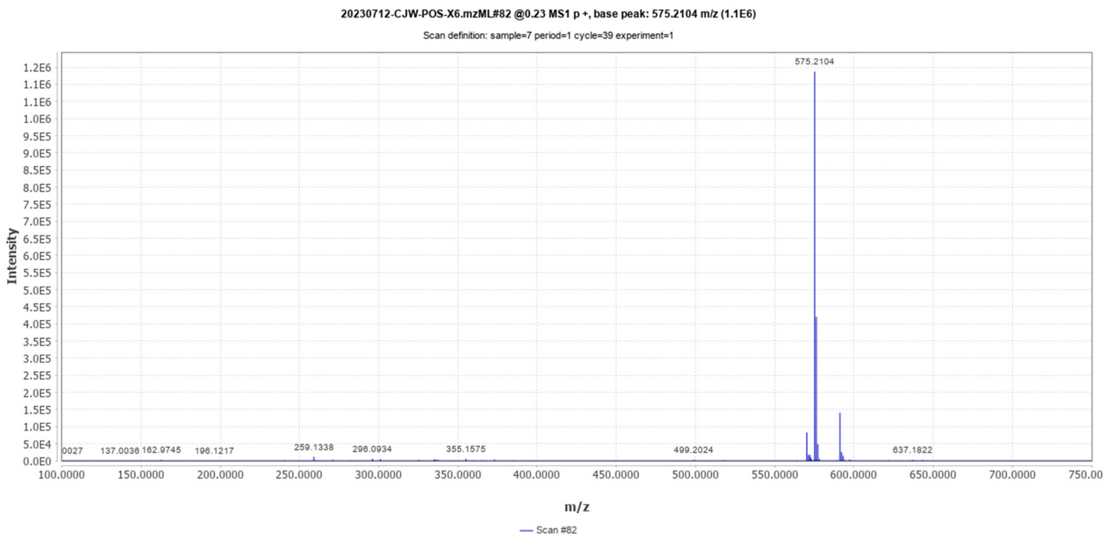

Figure S15. HRESIMS spectrum of compound **5**

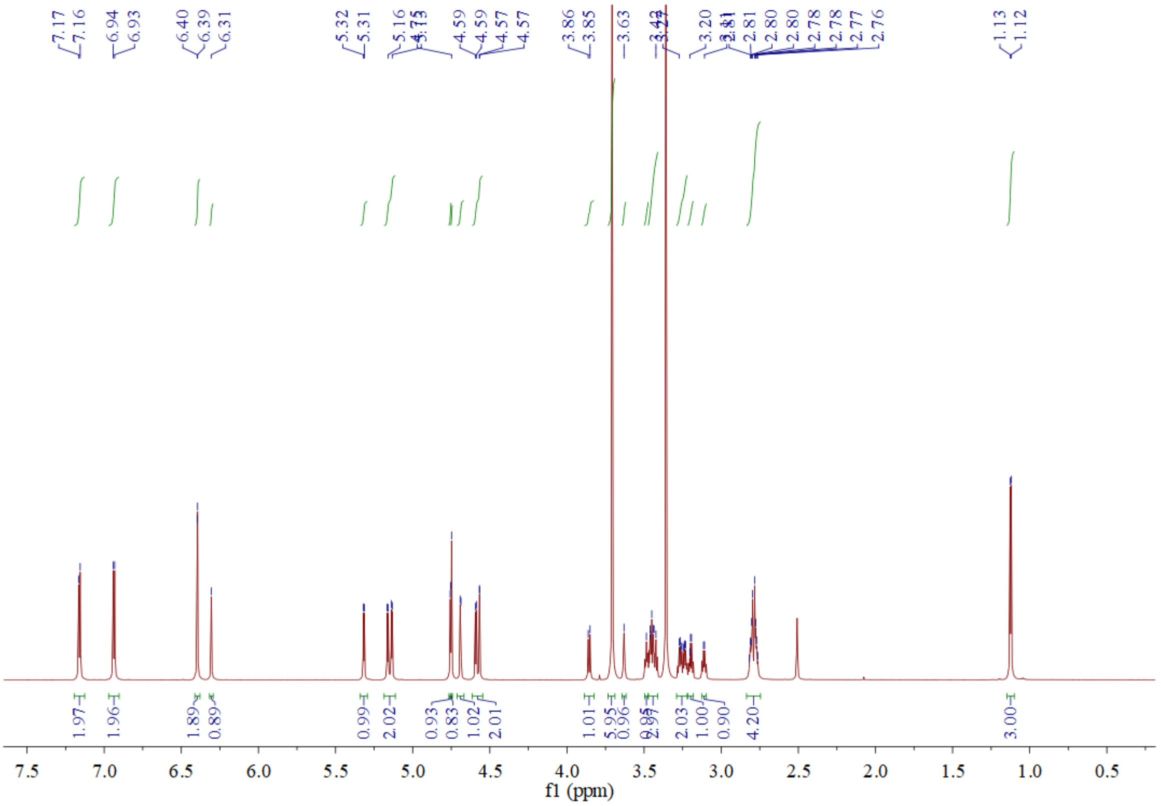

Figure S16. ^{1}H NMR spectrum (850 MHz) data of compound **6** in DMSO-d6

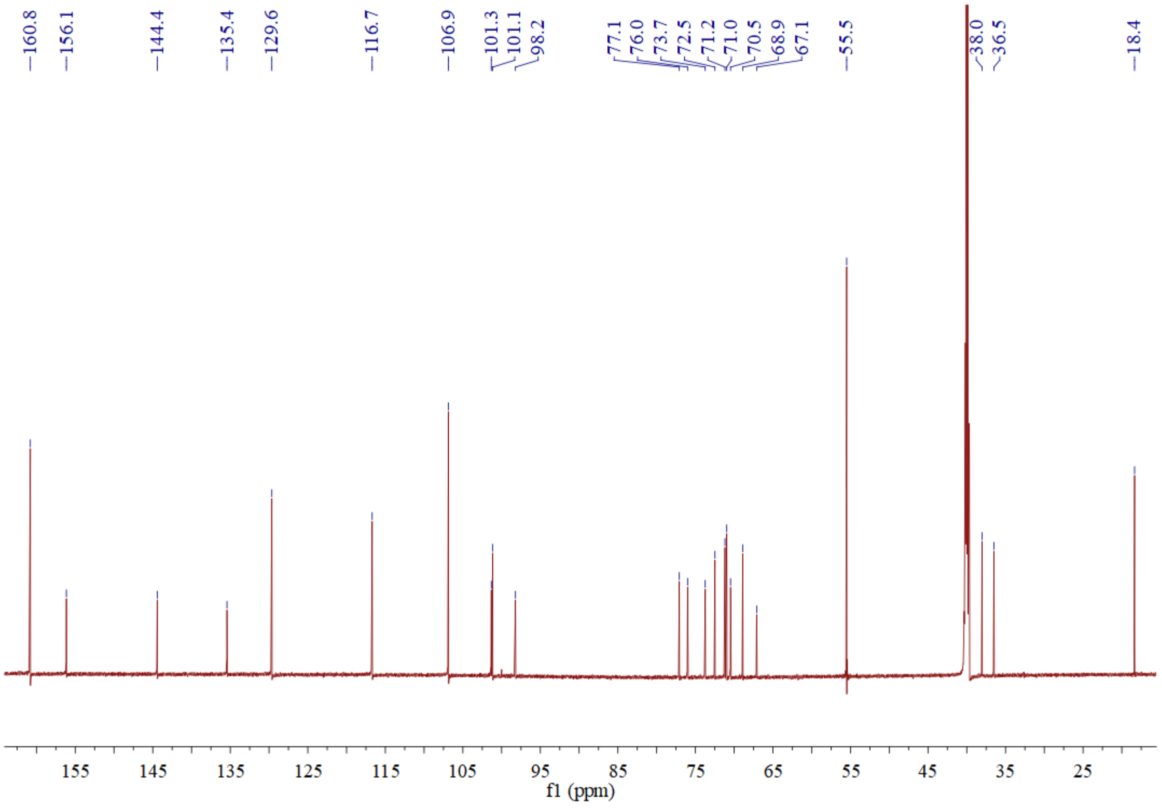

Figure S17. ^{13}C NMR spectrum (210 MHz) data of compound **6** in DMSO-d6

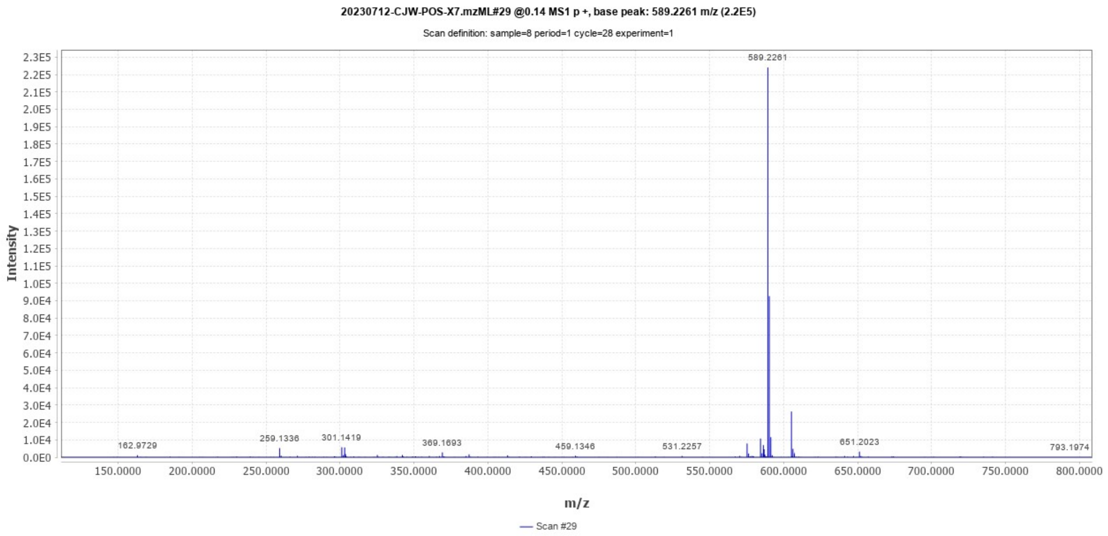

Figure S18. HRESIMS spectrum of compound **6**

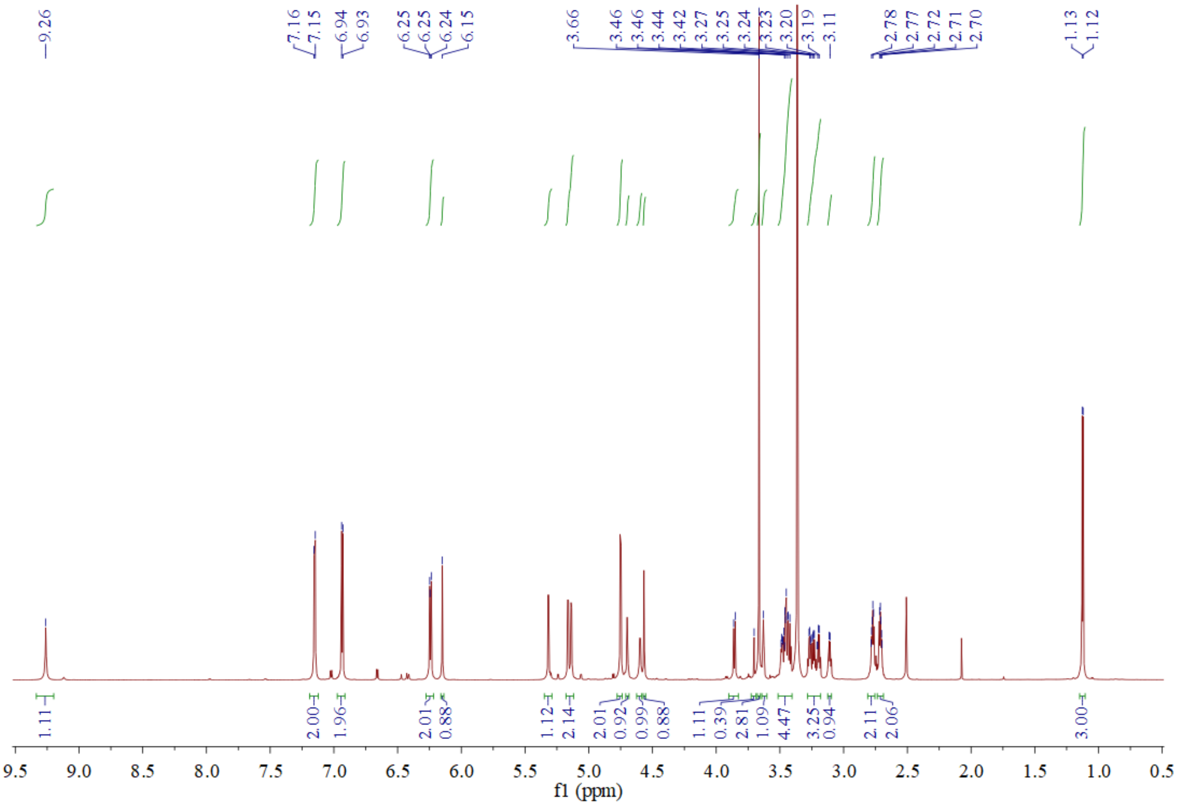

Figure S19. ^{1}H NMR spectrum (850 MHz) data of compound **7** in DMSO-d6

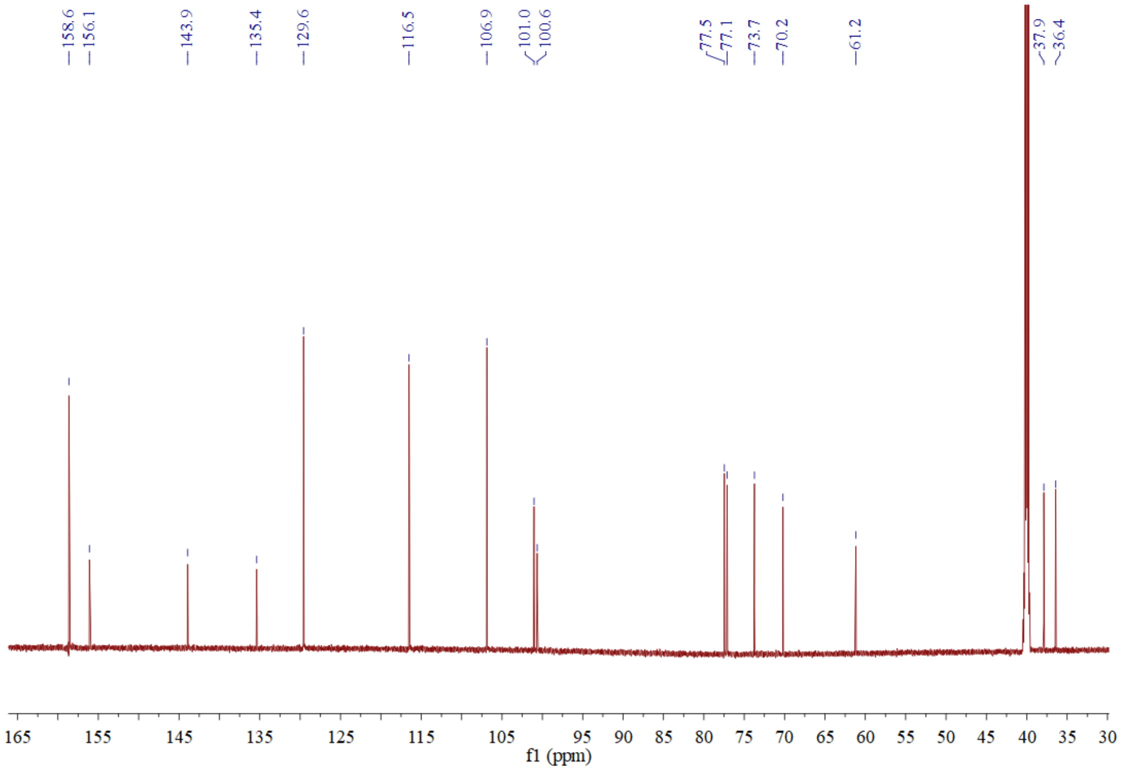

Figure S20. ^{13}C NMR spectrum (210 MHz) data of compound **7** in DMSO-d6

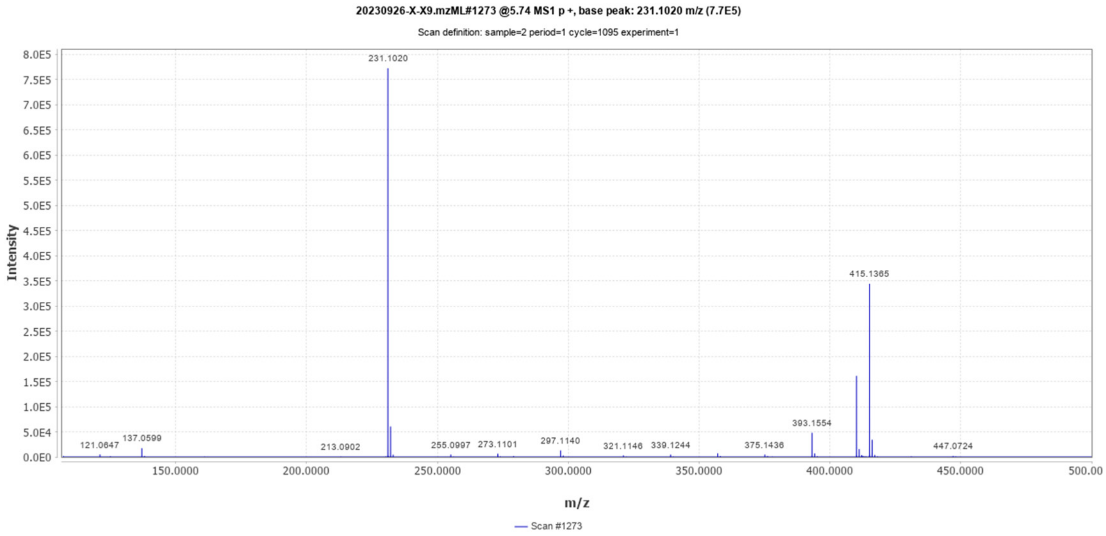

Figure S21. HRESIMS spectrum of compound **7**

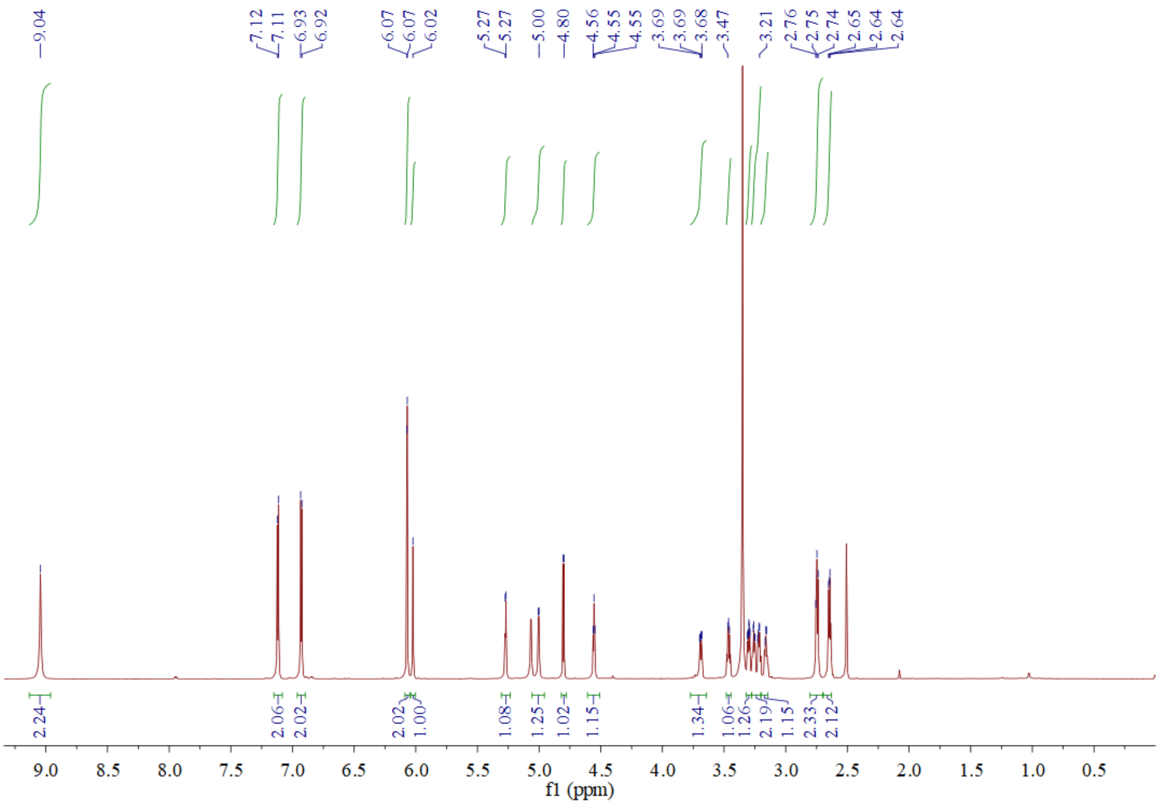

Figure S22. ^{1}H NMR spectrum (850 MHz) data of compound **8** in DMSO-d6

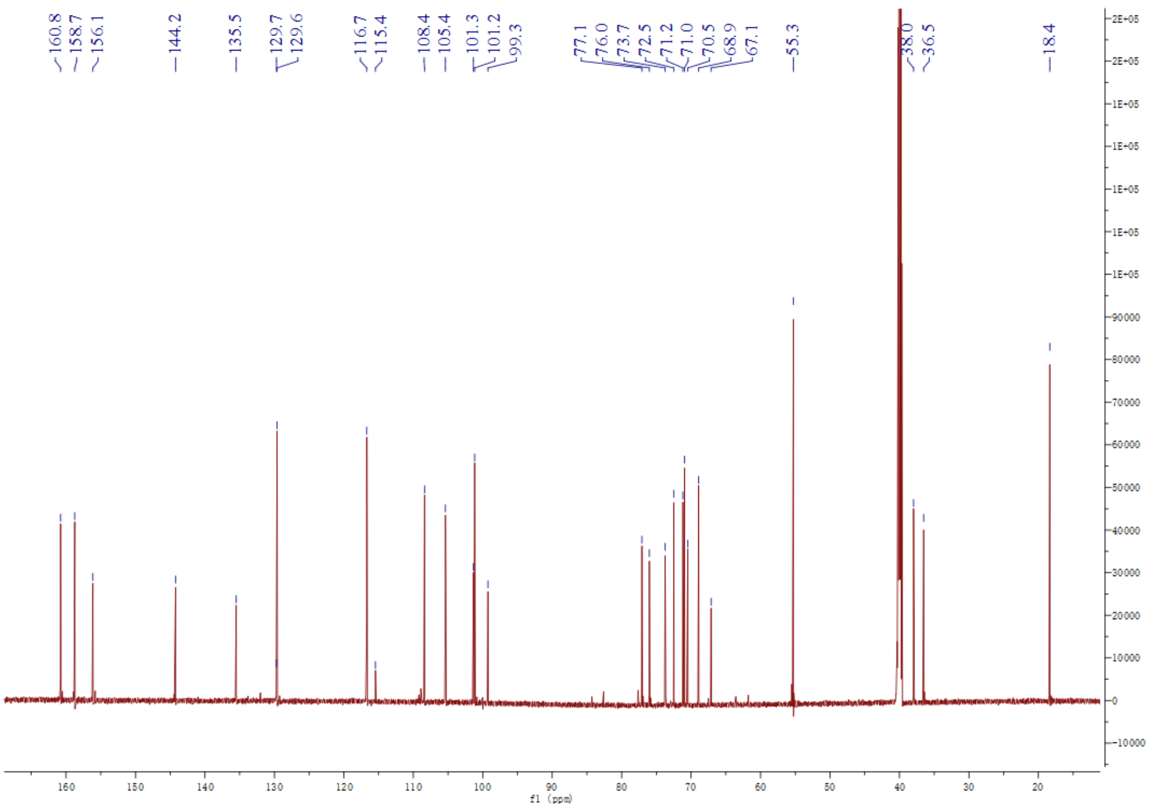

Figure S23. ^{13}C NMR spectrum (210 MHz) data of compound **8** in DMSO-d6

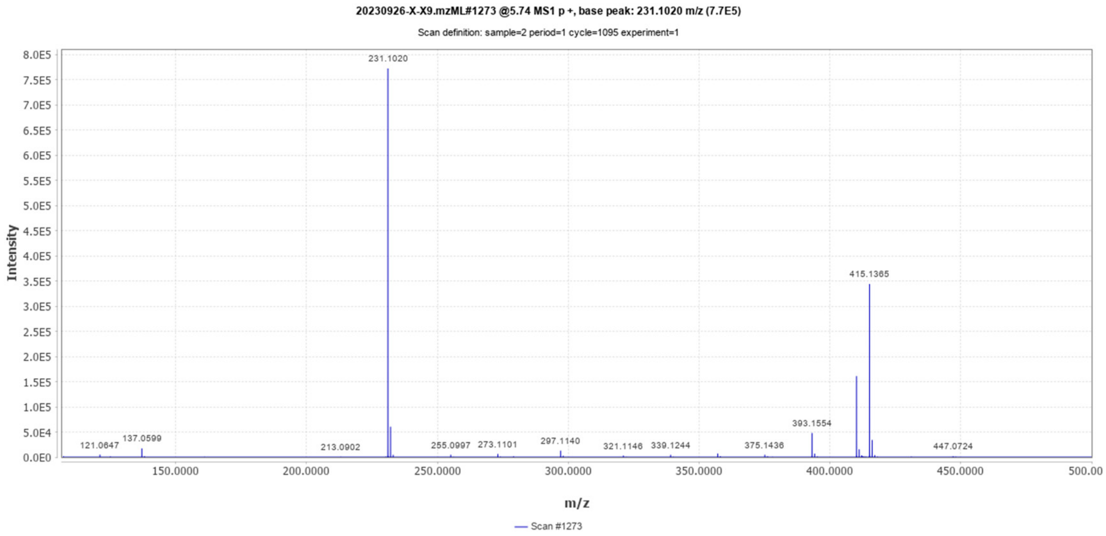

Figure S24. HRESIMS spectrum of compound **8**

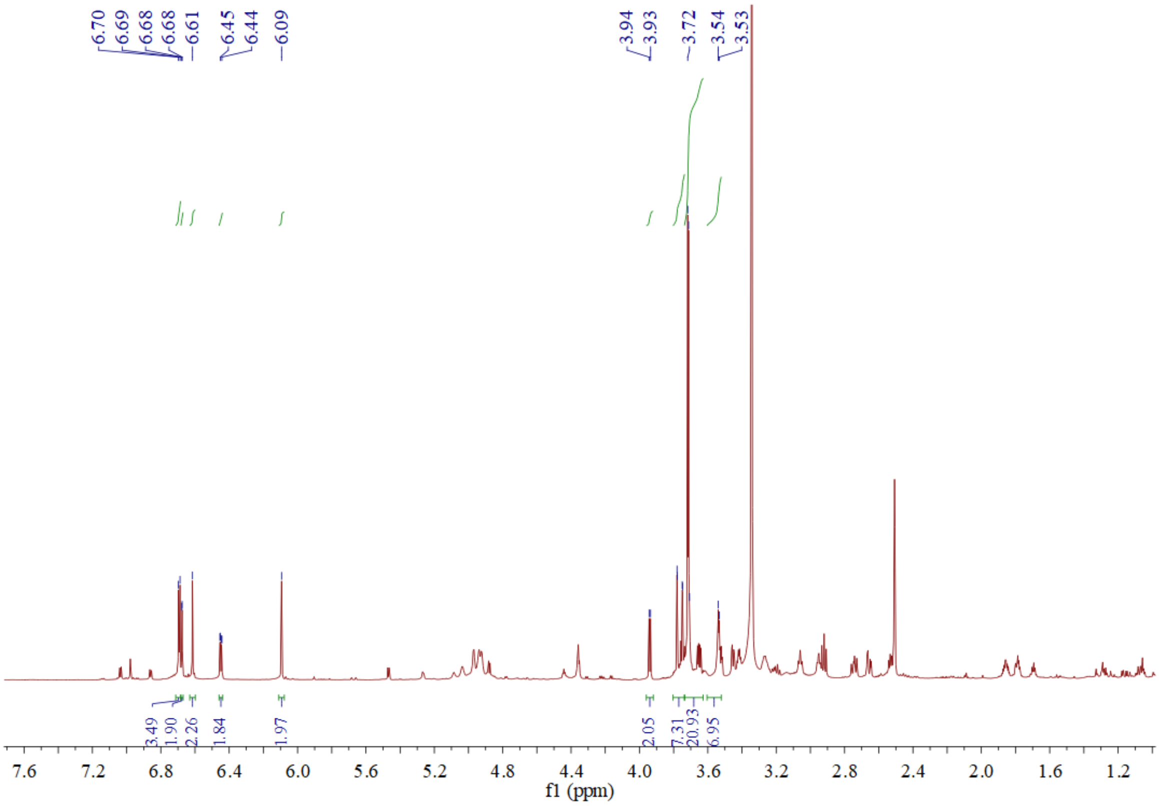

Figure S25. ^{1}H NMR spectrum (850 MHz) data of compound **9** in DMSO-d6

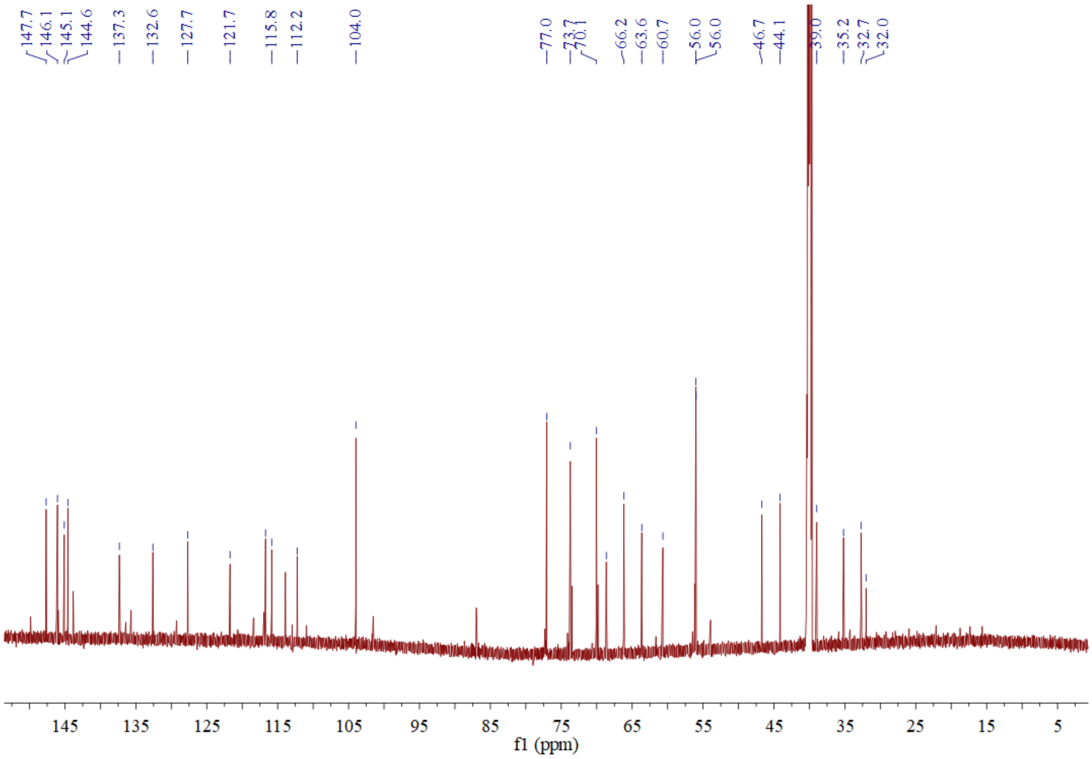

Figure S26. ^{13}C NMR spectrum (210 MHz) data of compound **9** in DMSO-d6

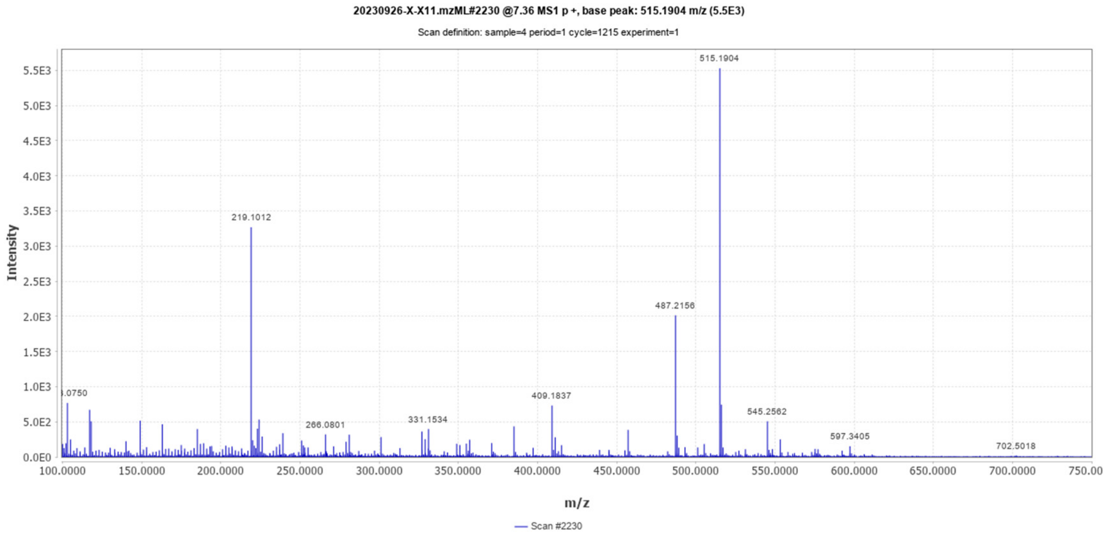

Figure S27. HRESIMS spectrum of compound **9**

References

1. Sun, M.; Wang, Y.; Han, X.; Yang, D.; Yang, J.; Ran, X.; Zhang, H.; Wang, T. Flavonoids from rhizomes of *Panax notoginseng* and their anti-platelet aggregation activity. *Chinese Traditional and Herbal Drugs*. **2025**, 56, 1500-1507.
2. Yang, Y.; Guo, T.; Xie, M.; Tan, L.; Li, W.; Zheng, H.; Huang, F.; Yang, Y.; Wang, W.; Jian, Y. Chemical constituents from the leaves of *Cyclocarya paliurus* and their alpha-glucosidase inhibitory activities. *Chinese Traditional Patent Medicine*. **2024**, 46, 834-842.
3. Shahat, A.A.; Abdel-Azim, N.S.; Pieters, L.; Vlietinck, A.J. Isolation and NMR spectra of syringaresinol-β-D-glucoside from *Cressa cretica*. *Fitoterapia*. **2004**, 75, 771-773, doi:10.1016/j.fitote.2004.05.008.
4. Chen, Y.; Tang, L.; Feng, B.; Shi, L.; Wang, H.; Wang, Y. New bibenzyl glycosides from leaves of *Camellia Oleifera* Abel. with cytotoxic activities. *Fitoterapia* **2011**, *82*, 481–484. https://doi.org/10.1016/j.fitote.2010.12.009.
5. Yang, D.; Cheng, Z.; Ding, Z.; Zhou, J.; Hu, J. Chemical constituents of Dendrobium crystallinum. *Guihaia*. **2017**, 37, 1182-1186.
6. Ito, T.; Ito, H.; Iinuma, M. Absolute configuration of resveratrol oligomer glucosides isolated from the leaves of *Upuna borneensis*. *Phytochem. Lett.* **2017**, 20, 26-31, https://doi.org/10.1016/j.phytol.2017.03.006.
7. Wang, J.; Lou, J.; Luo, C.; Zhou, L.; Wang, M.; Wang, L. Phenolic Compounds from *Halimodendron halodendron* (Pall.) Voss and Their Antimicrobial and Antioxidant Activities. *Int. J. Mol. Sci.* **2012**, 13, 11349-11364. https://doi.org/10.3390/ijms130911349.
